# HCMV pUS28 initiates pro-migratory signaling via activation of Pyk2 kinase

**DOI:** 10.1186/2042-4280-1-2

**Published:** 2010-12-07

**Authors:** Jennifer Vomaske, Susan Varnum, Ryan Melnychuk, Patricia Smith, Ljiljana Pasa-Tolic, Janani I Shutthanandan, Daniel N Streblow

**Affiliations:** 1 The Vaccine & Gene Therapy Institute, Oregon Health & Science University, Beaverton OR 97006 USA; 2 Pacific Northwest National Laboratories, Richland, WA 99352 USA

## Abstract

**Background:**

Human Cytomegalovirus (HCMV) has been implicated in the acceleration of vascular disease and chronic allograft rejection. Recently, the virus has been associated with glioblastoma and other tumors. We have previously shown that the HCMV-encoded chemokine receptor pUS28 mediates smooth muscle cell (SMC) and macrophage motility and this activity has been implicated in the acceleration of vascular disease. pUS28 induced SMC migration involves the activation of the protein tyrosine kinases (PTKs) Src and Focal adhesion kinase as well as the small GTPase RhoA. The PTK Pyk2 has been shown to play a role in cellular migration and formation of cancer, especially glioblastoma. The role of Pyk2 in pUS28 signaling and migration are unknown.

**Methods:**

In the current study, we examined the involvement of the PTK Pyk2 in pUS28-induced cellular motility. We utilized in vitro migration of SMC to determine the requirements for Pyk2 in pUS28 pro-migratory signaling. We performed biochemical analysis of Pyk2 signaling in response to pUS28 activation to determine the mechanisms involved in pUS28 migration. We performed mass spectrometric analysis of Pyk2 complexes to identify novel Pyk2 binding partners.

**Results:**

Expression of a mutant form of Pyk2 lacking the autophosphorylation site (Tyr-402) blocks pUS28-mediated SMC migration in response to CCL5, while the kinase-inactive Pyk2 mutant failed to elicit the same negative effect on migration. pUS28 stimulation with CCL5 results in ligand-dependent and calcium-dependent phosphorylation of Pyk2 Tyr-402 and induced the formation of an active Pyk2 kinase complex containing several novel Pyk2 binding proteins. Expression of the autophosphorylation null mutant Pyk2 F402Y did not abrogate the formation of an active Pyk2 kinase complex, but instead prevented pUS28-mediated activation of RhoA. Additionally, pUS28 activated RhoA via Pyk2 in the U373 glioblastoma cells. Interestingly, the Pyk2 kinase complex in U373 contained several proteins known to participate in glioma tumorigenesis.

**Conclusions:**

These findings represent the first demonstration that pUS28 signals through Pyk2 and that this PTK participates in pUS28-mediated cellular motility via activation of RhoA. Furthermore, these results provide a potential mechanistic link between HCMV-pUS28 and glioblastoma cell activation.

## Background

Human cytomegalovirus (HCMV) is a beta-herpesvirus able to establish a life-long persistent infection after primary infection has been cleared. Although infection is ubiquitous in the human population, persistent HCMV infection is commonly asymptomatic in healthy, immunocompetent individuals. However, HCMV causes significant disease in immunosuppressed patients and, despite effective antiviral therapies, HCMV infection is still a significant problem in congenital disease and in bone marrow transplant recipients. Additionally, HCMV has been associated with long-term diseases including the vascular diseases atherosclerosis, restenosis following angioplasty, and chronic allograft rejection following solid organ transplantation [1-4]. Recently, HCMV has been detected in human glioblastomas, and it has been suggested that HCMV exacterbates the progression of this disease [5]. Chemokines and their receptors have been identified as key mediators in chronic inflammatory processes that attend the development of vascular diseases and play a role in tumor development. Herpesviruses manipulate the host chemokine system by regulating the expression of host chemokines and chemokine receptors as well as by encoding chemokine and chemokine receptor homologues. Indeed, HCMV encodes a CXC chemokine (UL146), a putative CC chemokine (UL128), and four potential chemokine receptors (US27, US28, UL33 and UL78). pUS28 is one of the best characterized chemokine receptors and has been implicated in the development of long-term pathologies associated with HCMV infection, including vascular disease and malignancies [6-10].

pUS28 contains homology to the CC-chemokine receptors [11] and binds to a broad spectrum of CC-chemokine ligands, including RANTES/CCL5, MCP-1/CCL2, MCP-3/CCL7, and MIP-1β/CCL4. pUS28 is unusual in that it is able to bind the CX_3_C-chemokine Fractalkine/CX3CL1 with high affinity in addition to binding a variety of CC-chemokines [12,13]. Although the N-terminal 22 amino acids of pUS28 have been shown to be essential for binding of both chemokine classes, CC-chemokines fail to compete with CX3CL1 for receptor occupation. Mutagenesis studies of pUS28 indicate that different N-terminal residues are critical for binding to each chemokine class [12,14]. Chemokine binding to pUS28 initiates a variety of cellular signaling pathways [15-18]. Additionally, pUS28 has been shown to signal constitutively in the absence of ligand binding in several transformed cell lines [19]. pUS28 mediated migration of arterial SMC requires binding of the CC-chemokines CCL5 or CCL2 [20], while CX3CL1 inhibits pUS28-mediated SMC migration via the CC-chemokines [18]. However, CX3CL1 does actively promote the robust migration of pUS28 expressing macrophages suggesting that pUS28 has a dual function dependent upon the cell type it is expressed in and the local chemokine environment of that cell [18]. The binding of each chemokine ligand class (CC- vs. CX_3_C-) results in differential signaling at the level of G-protein coupling to pUS28 [18]. In fact, pUS28 binding to the CC-chemokines promotes SMC migration through the activation of Gα12/13 and the small G-protein RhoA. pUS28-mediated SMC migration can be blocked by expression of a dominant negative RhoA or RhoA effector associated kinase-1 (ROCK-1) [16].

pUS28 signals through the non-receptor tyrosine kinases focal adhesion kinase (FAK) and Src in a ligand-dependent manner, and this activity is required for induction of pUS28-mediated SMC migration. [17]. FAK is comprised of a central kinase domain flanked on one side by an N-terminal FERM (erythrocyte band 4.1-ezrin-radixin-moesin) domain, which is involved in linking FAK to integrins and growth factor receptors and provides regulation of the Tyr-397 autophosphorylation site [21,22]. Additionally, the FERM domain regulates FAK kinase activity via direct interaction with the kinase domain thereby blocking the access of substrates to the catalytic cleft [23]. The F.A.T. (focal adhesion targeting) domain is located C-terminal of the central kinase domain and is comprised of multiple protein-protein interaction motifs. FAK tyrosine phosphorylation following cellular stimulation is enhanced by its association with Src-family PTKs at Tyr-397. pUS28-mediated SMC migration is sensitive to the Src inhibitor PP2 and mutation of the FAK autophosphorylation site at Tyr-397 blocks SMC migration in a dominant-negative fashion. Conversely, a kinase-negative FAK mutant (FAK R454K) had no effect on pUS28-mediated SMC migration [17] suggesting that FAK acts as a protein scaffold rather than an active kinase in the signaling cascade leading to SMC migration.

Proline-rich Tyrosine kinase 2 (Pyk2), also a non-receptor tyrosine kinase, is highly related to FAK and plays an important role in cell motility [24]. Recently, Pyk2 has been shown to be involved in the CCR5-mediated chemotaxis of dendritic cells via binding of HIV-1 gp120 [25]. In addition, Pyk2 is critical for Angiotensin II (AngII)-mediated migration of vascular SMC (VSMC), in which Pyk2 mediates activation of RhoA and its effector kinase ROCK via phosphorylation of PDZ-Rho-GEF [26,27].

FAK and Pyk2 share an overall 45% sequence homology with 60% identity within the catalytic domain and also have analogous sites for tyrosine phosphorylation and Src binding [28]. Despite their striking sequence similarity, it is becoming increasingly clear that these molecules play very different roles in signaling cascades leading to cellular migration. Pyk2 is expressed in cells of the brain, hematopoetic cells, osteoblasts, some types of epithelium, SMC and fibroblasts [24]. Unlike FAK, Pyk2 activation has a significant dependence on intracellular calcium levels [28,29]. Calcium-mediated regulation of Pyk2 activity proceeds via binding of Ca2+/calmodulin to the Pyk2 FERM domain, allowing homodimerization and autophosphorylation at Tyr-402 [30]. Autophosphorylation of Pyk2 is believed to be a critical initial step in the activation of Pyk2 via the recruitment of Src to the phosphorylated Tyr-402 site, resulting in further tyrosine phosphorylation of Pyk2 [31,32]. However, Pyk2 activation can proceed by both Src-dependent [31,33-35] and Src-independent [36,37] mechanisms. Additionally, Pyk2-mediated phosphorylation of paxillin has been demonstrated to be independent of Tyr-402 autophosphorylation in SYF cells [32]. Collectively these data demonstrate that Tyr-402 autophosphorylation is not absolutely required for Pyk2 activity. While Pyk2 can partially compensate for the lack of FAK in fibroblasts derived from FAK-/- mouse embryos, Pyk2 alone is not sufficient to reconstitute a migratory phenotype in these cells [34]. Pyk2 and FAK interact in signaling events leading to cellular migration [38-41], but the relationship between these two proteins remains poorly defined and appears to be highly signal and cell context-specific Additionally, phosphorylation kinetics differ between the two proteins [37].

Here, we examined the involvement of Pyk2 in pUS28-induced signaling and SMC migration. Pyk2 is activated in SMC expressing pUS28 following the addition of ligand in a calcium-dependent manner. Expression of the autophosphorylation site mutant of Pyk2 (F402Y) blocks SMC migration in a dominant-negative fashion by preventing RhoA activation, while the kinase-inactive mutant has no effect on migration. Pyk2 forms an active kinase complex containing several novel Pyk2 binding proteins in CCL5 stimulated SMC expressing pUS28. Suprisingly, the autophosphorylation null mutant Pyk2 F402Y did not abrogate the formation of an active Pyk2 kinase complex but did prevent the pUS28-mediated activation of RhoA. Lastly, we demonstrate the pUS28-mediated activation of RhoA via Pyk2 also occurs in U373 glioblastoma cells. The Pyk2 kinase complex in U373 is distinct from the complex observed in RSMC and contains a number of proteins known to participate in glioma tumorigenesis. These results provide a potential mechanistic link between pUS28 signaling and glioma pathogenesis.

## Methods

### Cell Lines

The life-extended telomerized pulmonary artery SMC line, PAT1 [16] were maintained in Medium 199 supplemented with 20% fetal calf serum (FCS) and penicillin-streptomycin-L-glutamine (PSG; Gibco). For migration experiments described below, PAT1 cells were utilized between passage 5 and 30 post-telomerization. Mouse FAK-/- fibroblasts were maintained on gelatin coated culture dishes in Dulbecco's modified Eagle's Medium (DMEM) supplemented with 10% FCS, PSG, non-essential amino acids (Cellgro), and G418 (Sigma; 500 μg/ml) as previously described [34,42]. FAK-/- cells used in experiments were between passage 5 and 15. Primary F344 rat SMC (RSMC) were maintained in Dulbecco's modified Eagle's Medium (DMEM) with 10% FCS and PSG. RSMC were used between passage 5 and 20. U373 glioblastoma cells were maintained in DMEM supplemented with 10% FCS and PSG. U373 were used between passage 5 and 20.

### Reagents

Recombinant human CCL5 was purchased from R&D Systems and BAPTA-AM was from Sigma-Aldrich (A1076). Anti-Pyk2 (N-19), anti-phospho-402-Pyk2, anti-myc (9E10), anti-RhoA and anti-HA (F-7) antibodies were purchased from Santa Cruz Biotechnology. Secondary anti-mouse, anti-goat and anti-rabbit horseradish peroxidase (HRP)-conjugated antibodies were purchased from Amersham. Goat anti-mouse Alexa Fluor-488 was purchased from Molecular Probes (A11017).

### Adenovirus Construction

The adenovirus expressing pUS28-HA was previously described [20,43,44]. Myc-tagged Pyk2 WT, Pyk2 A457K (kinase inactive mutant) or Pyk2 F402Y (autophosphorylation mutant) [34] were constructed by cloning of the cDNA into pAdTet7, which contains the tet-responsive enhancer within a minimal CMV promoter followed by the SV40 late poly(A) cassette. Recombinant adenoviruses were produced by co-transfection of 293 cells expressing the Cre-recombinase with pAdTet7 constructs and adenovirus DNA (Ad5-Ψ5, an E1A/E3-deleted adenovirus genome) [45]. Recombinant adenoviruses were expanded on 293-Cre cells and the bulk stocks were titered on 293 cells by limiting dilution. Gene expression was driven by co-infection with an adenovirus expressing the Tet-off transactivator (Ad-Trans) [20].

### SMC migration assay

Cell migration assays were performed as previously described [20]. PAT1 cells were infected with HCMV (MOI 10) for 2 hrs followed by co-infection with Ad-Trans and Ad-Pyk2 (WT, A457, F402Y) at MOI of 50 for an additional 2 hrs. Subsequently, the transwells were transferred to new 12-well plates containing fresh medium with CCL5 (50 ng/ml) added to the lower chamber. Cells migrating into the lower chamber were counted at 48-72 hours post-infection (hpi) using a Nikon TE300 microscope at magnification 10×. Experiments were performed in at least triplicate wells and ten random fields were read in each well. The average number of cells per well was determined by multiplying the average number of cells per 10× field by the number of fields per well. Mean and standard deviation were calculated. Pyk2 recombinant protein levels were monitored by western blotting and equalized by adjusting the adenoviral vector MOI accordingly.

### Immunofluorescence

Subcellular localization of adenovirus-expressed proteins was visualized by fixing adenovirus-infected SMC in 4-well chamber slides (Nunc Lab-Tek) at 24 hpi in 2% paraformaldehyde for 15 min. Cells were then permeablized with PBS containing 0.2% Saponin and 0.02% BSA (sapPBS) for 15 min. Anti-myc primary antibody was diluted 1:500 in sapPBS and cells were incubated for 1 hr at room temperature (r.t.), washed twice with sapPBS, and incubated with goat anti-mouse Alexa Fluor-488 diluted 1:1000 in sapPBS for 30 min at r.t. After two more washes in sapPBS cells were visualized on a Deltavision deconvolution fluorescence microscope (Applied Precision).

### Pyk2 Y402 Phosphorylation Assay

FAK-/- or U373 cells plated onto 6-well culture dishes at 50% confluence were serum-starved for 6 hrs. The cells were infected with Ad-Trans and Ad-pUS28 at MOI 150 with Ad-Pyk2 or Ad-Pyk2 F402Y at MOI 50 and placed in serum free medium. For calcium chelation assays, cells were pre-treated with 50 μM BAPTA-AM for 30 min prior to stimulation. At 16 hpi, the cells were stimulated with CCL5 (40 ng/ml) and then scraped in 2× Laemmeli's Sample Buffer. Unboiled samples were loaded on 10% SDS-PAGE. The gels were transferred to Immobilon-P membranes then blocked in PBS containing 3% milk + 0.1% Tween 20 for 15 min at r.t. The primary anti-Phospho-Y402 Pyk2 rabbit polyclonal antibody was added at 1:1000 dilution in blocking buffer for 1 hr at r.t. The blots were washed with TBS-Tween buffer (10 mM Tris pH 7.2, 100 mM NaCl, 0.2% Tween-20). The secondary antibody (goat anti-rabbit conjugated to HRP) was added at 1:20,000 dilution in blocking buffer for 30 min at r.t. After washing 3 times with TBS-Tween buffer and incubation with ECL reagents, the blots were visualized by autoradiography. Blots were dried to inactivate the HRP secondary reagent and then reprobed as above with anti-Pyk2 goat polyclonal antibody at 1:1000 dilution in blocking buffer followed by secondary donkey anti-goat conjugated to HRP at 1:100,000 dilution.

### RhoA Activation Assay

FAK-/- or U373 cells plated onto 6-well culture dishes at 50% confluence were serum-starved for 6 hrs. The cells were infected with Ad-Trans only or co-infected with Ad-Trans and Ad-pUS28 at MOI 150 with or without Ad-Pyk2 or Ad-Pyk2 F402Y at MOI 50 and placed in serum free medium. After 16 hrs, the cells were stimulated with CCL5 (40 ng/ml) and then scraped in equal volumes modified RIPA buffer (10 mM Tris pH7.4, 150 mM NaCl, 1 mM EDTA, 1% Triton X-100, 1% Sodium Deoxycholate, 0.1% SDS) containing protease inhibitor cocktail (100 mM AEBSF, 80 μM aprotinin, 1.5 mM E-64, 2 mM leupeptin hemisulfate, and 1 mM pepstatin A) at 0 (unstimulated), 2.5, 5, or 10 minutes post-stimulation. Cell lysates were homogenized via sonication, then incubated with 20 μl Rho Assay Reagent (Rhotekin-RBD-GST Agarose; Upstate) for 1 hr at 4°C, then washed 3 times with 1 ml of RIPA. The final bead pellet was resuspended in 40 μl of 2× Laemmli's sample buffer, boiled and then run on 10% SDS-PAGE. The gels were transferred to Immobilon-P membranes then blocked in blocking buffer (PBS containing 3% Milk + 0.1% Tween 20) for 15 min at r.t. The primary anti-RhoA mouse monoclonal antibody was added at 1:1000 dilution in blocking buffer for 1 hr at r.t. The blots were washed with TBS-Tween buffer. The secondary antibody (goat anti-mouse conjugated to HRP) was added at 1:40,000 dilution in blocking buffer for 30 min at r.t. After washing 3 times with TBS-Tween buffer and incubation with ECL reagents, the blots were visualized by autoradiography.

### Pyk2

In Vitro *Kinase Assays. In vitro *kinase assays were performed on on immunoprecipitated Pyk2 from F344 aortic RSMC. Cells were plated in 10 cm culture dishes and serum starved for 24 hrs. The RSMC were co-infected with Ad-Trans and Ad-Pyk2 or Ad-Pyk2 F402Y at an MOI of 50 with or without Ad-pUS28 at an MOI of 150. After 16 hrs, the cells were stimulated with recombinant human CCL5 (40 ng/ml) and then harvested at times 0 (unstimulated), 2.5, 5, 10, and 15 minutes post ligand addition. Cells were rinsed in PBS and lysed in modified RIPA containing protease inhibitor cocktail and 200 μM sodium orthovanadate. Total Pyk2 was immunoprecipitated using mouse anti-myc tag monoclonal antibody and Protein-A/G conjugated agarose beads (Santa Cruz Biotechnology). Precipitation reactions were washed one time in Triton-only lysis buffer (modified RIPA without sodium deoxycholate and SDS), two times in HNTG buffer (50 mM HEPES, 150 mM NaCl, 1% Triton, 10% Glycerol, pH 7.4), and two times in kinase buffer (20 mM HEPES, 10 mM MgCl_2_, 10 mM MnCl_2_, 150 mM NaCl, 10% Glycerol pH 7.4) and then resuspended in 50 μl kinase buffer plus 10 μCi ^32^P-γ-ATP (Perkin-Elmer). The kinase reaction was allowed to proceed for 30 minutes at r.t. then stopped by the addition of 2× Laemmeli's sample buffer. After boiling for 5 minutes, the reactions were run on 10% SDS-PAGE and transferred to Immobilon-P membranes. The incorporation of ^32^P-g-ATP was visualized via autoradiography. After allowing radioactivity to decay, the membranes were further analyzed via western blot as above for the presence of Pyk2-myc and pUS28-HA.

### Preparation of Pyk2 Complexes for Proteomics Analysis

Proteomics analysis was performed on immunoprecipitated Pyk2 from F344 aortic RSMC or U373 glioblastoma cells. Cells were plated in 15 cm culture dishes. The RSMC were serum starved for 24 hrs prior to co-infection with Ad-Trans and Ad-Pyk2 at an MOI of 50 with or without Ad-pUS28 at an MOI of 150. After 16 hrs, the cells were stimulated with recombinant human CCL5 (40 ng/ml) and then triplicate dishes were harvested at times 0 (unstimulated), 5, 10, 15, 30 and 60 minutes post ligand addition. Cells were rinsed in PBS and lysed in modified RIPA containing protease inhibitor cocktail and 200 μM sodium orthovanadate. Total Pyk2 was immunoprecipitated using mouse anti-myc tag monoclonal antibody and Protein-A/G conjugated agarose beads (Santa Cruz Biotechnology). Precipitation reactions were washed twice with RIPA. Pyk2 and associated proteins were eluted with 200 μl of Pierce Gentle Ag/Ab Elution buffer. Triplicate eluates were combined and the samples were sequenced by tandem mass spectrometry analysis at the EMSL user facility at Pacific Northwest National Laboratories.

### Tryptic digestion of Pyk2 Complexes

Proteins from the Pyk2 complexes were desalted and concentrated with Amicon ultra filtration units (Millipore, Billerica, MA). Protein concentration was determined using BCA protein assay (Thermo Scientific) and the concentrated protein was denatured by the addition of trifluoroethanol (TFE) to a final concentration of 50% and heating to 37°C for 60 min. Denatured proteins were reduced with DTT (2 mM final concentration) and diluted fivefold with 50 mM NH4HCO3. Methylated, sequencing-grade porcine trypsin (Promega, Madison, WI) was added at a substrate-to-enzyme ratio of 50:1 (mass to mass) and incubated at 37°C for 3 hrs. The peptides were concentrated with a speed vac and stored at 80°C until analysis.

### Tandem mass spectrometric analysis of peptides

Peptide samples were analyzed using an automated custom built capillary LC system containing a four capillary column system (Livesay et al, Anal Chem 2008, vol 80, page 294). Eluate from the LC was coupled directly to a hybrid linear ion-trap-orbitrap (LTQ_Orbitrap, Thermo Electron Corp.). The reverse-phase capillary column was prepared by slurry-packing 3-micron Jupiter C18 bonded particles (Phenomenex, Torrence, CA) into a 65 cm long, 75-micron-inner diameter fused silica capillary (Polymicro Technologies, Phoenix, AZ). After peptide loading onto the column, the mobile phase was held at 100% A (0.05% trifluoroacetic acid (TFA) and 0.2% acetic acid in water) for 20 min, followed by a linear gradient from 0 to 70% buffer B (0.1% TFA in 90% acetonitrile, 10% water) over 80 min with a flow rate of ~500 nL/min. Orbitrap spectra (AGC 1 × 10^6^) were collected from 400-2000 m/z at a resolution of 100 k followed by data-dependent ion trap tandem mass spectra (AGC 1 × 10^4^) of the six most abundant ions using a collision energy of 35%. The heated capillary was maintained at 200°C, and the ESI voltage was held at 2.2 kV.

### SEQUEST analysis

The SEQUEST algorithm was run on each of the datasets against the human.fasta from the International Protein Index database (version 3.54, 75,419 entries, released January 2009). MS/MS peaks were generated by "extract_msn.exe," part of the SEQUEST software package. A peptide was considered to be a match by using a conservative criteria set developed previously by Yates and coworkers [45]. Briefly, peptides were retained if they met the following criteria: 1). SEQUEST DelCN value of ≥0.1 and 2). SEQUEST correlation score (Xcorr) > 1.9 for charge state 1+ and fully tryptic; Xcorr > 2.2 for charge state 2+ and fully or partially tryptic; Xcorr > 3 for charge state 2+ regardless of tryptic state; Xcorr > 3.75 for charge state 3+ for fully or partially tryptic.

## Results

### Pyk2 Autophosphorylation is Required for pUS28-mediated SMC Migration

**Figure 1:**
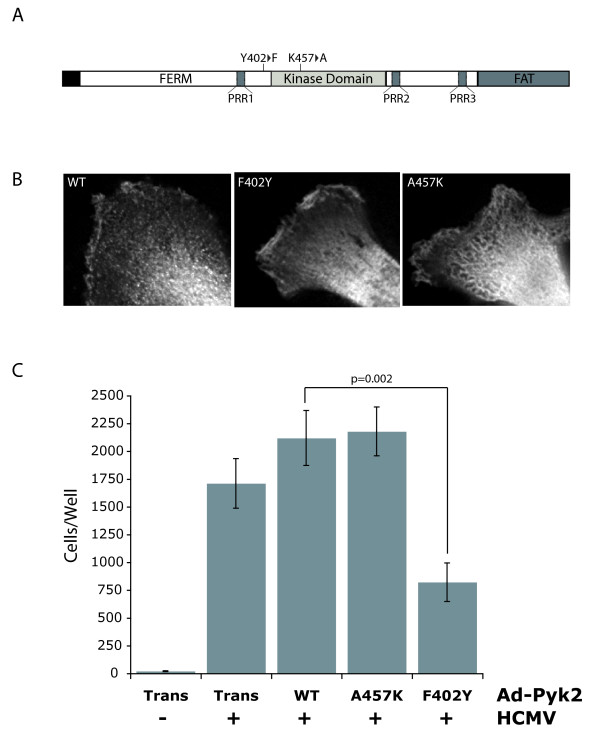
**Pyk2 Autophosphorylation Mutant Inhibits HCMV-mediated SMC**. (A) Schematic of Pyk2 domain structure with A457K and F402Y mutations annotated. (B) Sub-cellular localization of myc-tagged adenovirus constructs was determined via indirect immunofluorescence staining for the myc epitope. From left Ad-Pyk2 WT, Ad-Pyk2-F402Y and Ad-Pyk2-A457K in lamellipodia of adenovirus-infected PAT1 SMC. Gene expression was driven my co-infection with an adenovirus expressing the Tet-off transactivator (Ad-Trans) (C) SMC migration assays were performed on PAT1 SMC co-infected with HCMV at MOI 10 and adenoviruses expressing control Ad-Trans only or Ad-Trans+Pyk2-WT, Pyk2-A457K, or Pyk2-F402Y at MOI 50 in transwell chambers. At 2 hrs post-infection fresh medium containing 50 ng/ml CCL5 was added to the bottom chamber and cells were allowed to migrate for 48 hrs. Migrated cells in the lower chamber were counted via light microscopy. For each condition, n = 6 from two independent experiments.

In order to establish the involvement of Pyk2 in pUS28-mediated SMC migration, we constructed a panel of adenovirus vector expressing myc-tagged versions of wild-type Pyk2 (Pyk2-WT), an autophosphorylation null mutant (Pyk2-F402), and a kinase-inactive mutant (Pyk2-A457) (Figure 1A) [34]. These Pyk2 mutant adenoviruses were expressed in human pulmonary artery smooth muscle (SMC) cells. Gene expression was driven by co-infection with the Tet-off transactivator adenovirus (Ad-Trans) and Ad-Trans infection alone is used to control for any non-specific effects of adenovirus infection. All Pyk2 constructs displayed similar cellular distribution patterns in the cytoplasm and at lamellipodia of growing cells (Figure 1B). As previously described, human SMC cells infected with HCMV migrated in response to CCL5 [20,46] (Figure 1C). Expression of Pyk2-WT produced similar levels of migration compared to Ad-Trans only controls. Surprisingly, co-infection with adenovirus producing kinase-inactive Pyk2 (Pyk2-A457) did not abrogate HCMV-mediated SMC. In contrast, expression of a mutant Pyk2 that lacks the Tyr-402 autophosphorylation site (Pyk2-F402) acted as a dominant negative inhibitor of HCMV-mediated SMC migration, causing a 60% reduction in SMC migration compared to HCMV-infected SMC expressing Pyk2-WT (Figure 1C). These results demonstrate that Pyk2 kinase activity is dispensable for pUS28-mediated SMC migration and suggest that the primary role of Pyk2 in the pro-migratory signaling cascade in these cells is to act as a signaling scaffold for the activation of Src kinase [31].

### pUS28 Signaling leads to Phosphorylation of Pyk2 at Y402

In order to further characterize the role of Pyk2 in pUS28-mediated cellular migration, we examined phosphorylation of Pyk2 at Y402 in Ad-pUS28 infected mouse fibroblasts lacking FAK (FAK-/-). FAK-/- cells are known to have elevated levels of endogenous Pyk2, partially compensating for the lack of FAK [34,47]. We have utilized FAK-/- for pUS28 signaling assays in previous studies [17,18]. FAK-/- exhibit low baseline activation of pro-migratory signaling molecules and are therefore a highly inducible system in which to study these signaling events. Furthermore, using this system we can study pUS28 signaling to Pyk2 independent of FAK.

**Figure 2:**
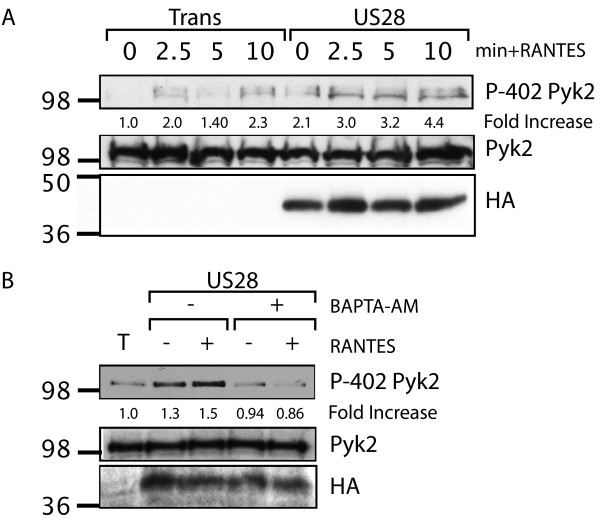
**pUS28 Signaling Causes Calcium-Dependent Phosphorylation of Pyk2 at Y402**. (A) FAK-/- fibroblasts were infected with Ad-Trans only or Ad-Trans+Ad-pUS28 for 18 hrs. Cells were stimulated with 40 ng/ml CCL5 for the indicated times and analyzed via western blot with a phospho-specific Pyk2-Y402 antibody or total Pyk2 antibody. (B) Adenovirus-infected FAK-/- were pre-treated for 30 min with BAPTA-AM to chelate intracellular calcium and then stimulated with 40 ng/ml CCL5 for 5 min. Total cell lysates were analyzed via western blot for phospho-Y402 Pyk2 and total Pyk2. For both experiments, blots were stripped and reprobed to verify pUS28-HA expression. Phospho-specific blots were quantified via densitometry using ImageJ software and are expressed as fold change compared to unstimulated, Ad-Trans infected control.

pUS28-expressing FAK-/- stimulated with CCL5 showed sustained phosphorylation of Pyk2 at Y402. Levels of Pyk2 phosphorlyation were ~2-fold higher in the presence of pUS28 than control Ad-Trans infected cells similarly stimulated. CCL5 stimulation had a small but quantifiable effect on Pyk2 phosphorylation in FAK-/- cells infected with Trans only, we believe this activation is attributable to the effects of calcium gradients produced by the addition of media upon stimulation (Figure 2A). Pyk2 kinase activity has been shown to be dependent on intracellular calcium levels [28-30]. Therefore, we treated pUS28-expressing FAK-/- with BAPTA-AM to chelate intracellular calcium prior to performing signaling assays. The cells were then stimulated with CCL5 and examined for Pyk2 Y402 phosphorylation via western blot. As expected, chelation of intracellular calcium abrogated phosphorylation of Pyk2 in response to pUS28 signaling (Figure 2B) and prevented Pyk2 activation in Ad-Trans controls (data not shown). We observed a low, but measurable, effect of US28 expression on Pyk2 activity in the absence of ligand (Figure 2A and 2B), which could be attributed to either low-level constitutive signaling or minor effects of endogenously expressed ligands.

### pUS28 Signaling Causes formation of a Pyk2-Associated Active Kinase Complex

**Figure 3:**
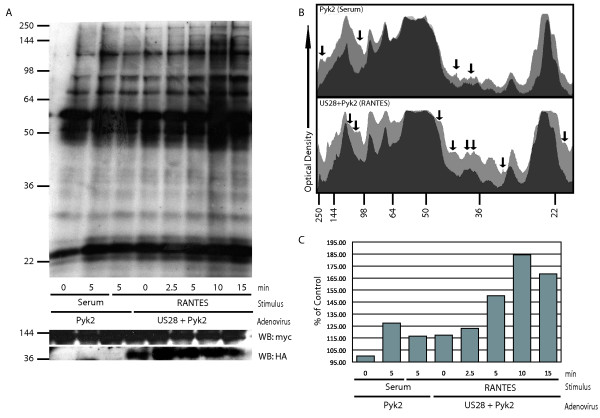
**pUS28 Signaling Causes Formation of an Active Kinase Complex Involving Pyk2**. SMC were infected with Ad-Trans and Ad-Pyk2 +/- Ad-pUS28 and stimulated with 20% serum or 40 ng/ml CCL5 for indicated times. Pyk2 was immunoprecipitated using myc antibodies. *In vitro *kinase reactions were performed on immunoprecipitated material and reactions were loaded on SDS-PAGE, transferred to immobilon-P membranes and visualized via autoradiography. (A) Autoradiogram of *in vitro *kinase reactions. Total Pyk2 and pUS28 expression was determined by western blot for myc and HA tags, respectively. (B) Densitometric lane plots of results shown in panel A, generated using ImageJ software. The darker curve (overlay) is unstimulated and the lighter curve (background) is 5 min post-stimulation for Pyk2 only stimulated with serum (top) or Pyk2+pUS28 stimulated with CCL5 (bottom). Black arrows indicate bands present only in stimulated samples. Molecular weight markers are shown on the density plot to facilitate comparison to the autoradiogram in panel A. (C) Overall optical density quantification of each lane in Pyk2 *in vitro *kinase reactions shown in panel A. Values are displayed as percentages compared to unstimulated samples infected with Ad-Trans+Ad-Pyk2.

We have shown that phosphorylation of Pyk2 at Y402 is required for SMC migration (Figure 1C) and that pUS28 signaling causes phosphorylation at this critical site independent of any pUS28 activation of FAK (Figure 2A). Although activation at the autophosphorylation site is important for Pyk2 function [32], we wanted to further characterize the effect of pUS28 signaling on the activation and function of Pyk2. To accomplish this, we performed *in vitro *kinase assays on Pyk2 immunoprecipitation reactions. For these experiments we returned to examining Pyk2 signaling in the context of SMC. However, in human SMC endogenous production of pUS28 ligands obscures signaling activation [48]. Therefore, as in previous studies [18], we chose to perform these experiments in primary rat SMC (RSMC) to eliminate this background signaling. Cells were serum starved and co-infected with adenovirus vectors expressing Pyk2-WT with or without pUS28-HA. The cells were then stimulated with either 20% serum, as a positive control for Pyk2 activation, or 40 ng/ml CCL5 and harvested at 0, 2.5, 5, 10 or 15 min post-stimulation. Pyk2 was then immunoprecipitated from total cell lysates using a monoclonal antibody directed against the myc tag and immunoprecipitated proteins were subjected to *in vitro *kinase assay. The Pyk2 kinase activity was analyzed via SDS-PAGE and autoradiography. pUS28 signaling promoted the formation of a large kinase complex involving Pyk2 and a number of other interacting phosphoproteins. This protein complex begins to form by 2.5 min after the addition of CCL5 peaking at 10 min post ligand treatment (Figure 3A and 3C). Plotting the optical density of unstimulated versus 5 min stimulated timepoints using ImageJ software revealed several pUS28-specific proteins associated with Pyk2 (Figure 3B). When a parallel experiment was performed in the presence of Pyk2-F402Y, a similar kinase complex was observed (data not shown), indicating that Tyr-402 is not critical for formation or maintenance of Pyk2 in an active kinase complex but rather mediates the binding of critical signaling elements necessary for the Pyk2 scaffold to participate in pro-migratory signaling.

### Identification of Pyk2 Binding Partners

**Figure 4:**
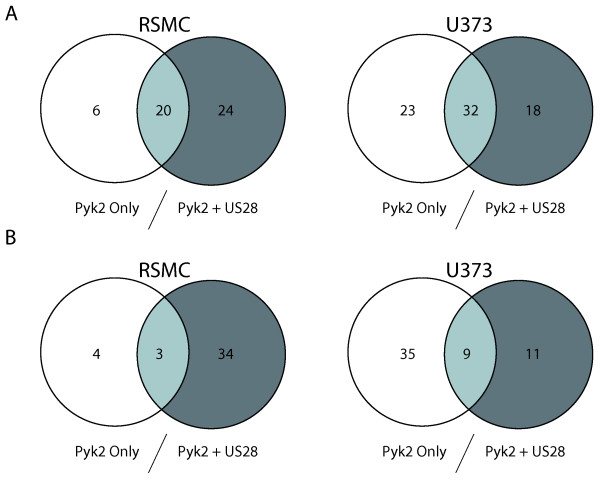
**Pyk2-Associated Proteins are Cell-Type and Signal-Specific**. SMC or U373 were infected with Ad-Trans and Ad-Pyk2 +/- Ad-pUS28 and stimulated with 20% serum or 40 ng/ml CCL5 for 0, 5, 10, 15, 30 or 60 min. Pyk2 was immunoprecipitated using myc antibodies. Pyk2 complexes were analyzed via tandem mass spectrometry. Results shown are proteins identified with >3 spectral hits for all peptides. (A) Total hits for Pyk2-associated proteins in RSMC and U373. (B) Proteins for which Pyk2-association was induced over the timecourse of stimulation in RSMC and U373. For data analysis, a protein was considered induced if the average spectral hits for all peptides for timepoints 5, 10, 15, 30 and 60 min was greater than twice the spectral hits for all peptides in unstimulated cells.

We performed proteomics analysis on Pyk2 kinase complexes in order to determine whether pUS28 signaling promotes the association of Pyk2 with novel binding partners. We isolated Pyk2 complexes via immunoprecipitation as described above from RSMC expressing pUS28-HA and Pyk2 at 0, 5, 10, 15, 30 or 60 min post-addition of CCL5. As a control for the pUS28-specificity of Pyk2 interactions, we performed identical immunoprecipitation reactions in RSMC expressing Pyk2 only stimulated with 20% serum. Bound proteins were analyzed via tandem mass spectrometry. We identified a total of 50 Pyk2 binding partners in RSMC and 24 of these associated with Pyk2 specifically in response to pUS28 expression. Only 6 proteins were specific for Pyk2 in the absence of pUS28 (Figure 4A Table 1 Additional File 1). When we examined the proteins whose Pyk2 association was specifically induced over the signaling timecourse, we determined that 34 proteins associated with Pyk2 in response to pUS28 stimulation by CCL5. In contrast, only 4 proteins were specifically activated by serum stimulation of RMSC expressing Pyk2 only (Figure 4B). As expected, we identified a number of cytoskeleton structural proteins in our proteomics screen confirming that Pyk2 is directly associated with the cytoskeleton when activated [49] (Table 1 and Additional File 1). We identified several proteins known to interact with Pyk2 in other systems including calmodulin [50] and gelsolin [51]. In support of our hypothesis that Pyk2 serves as a scaffold for pUS28 signaling in RSMC, we found several signaling intermediates associating with Pyk2 over the timecourse. These included a RhoGEF (Trio) known to link G-protein signaling to RhoA activation [52], a regulator of MAPK signaling (KSR-2) [53] as well as a regulator of the SHP-1 tyrosine phosphatase (TFG) [54]. Interestingly, we observed a pUS28 inducible association of Pyk2 with the NFκB precursor p105. Although Pyk2 signaling has been implicated in NFkB activation [55,56], we believe that our data showing NFκB p105 present in an activated Pyk2 complex is novel in the literature. This interaction may provide a secondary, ligand-dependent mechanism for pUS28-mediated activation of NFκB.

**Table 1:** Pyk2 binding partners in pUS28-expressing RSMC

CATEGORY	PROTEIN(S) IDENTIFIED
**Cytoskeleton Structure and Dynamics**	β-actin, α1-actin, F-actin capping proteins-α1, -α2*, and -β, Gelsolin [51,72,88,89], Myosin-9, -10, -11, Myosin light chain-6, Tropomodulin-3[72], Tropomyosin-α1, α3, α4 -β [72,90,91], Tubulin-β-2C, Vimentin [92]

**Signal Transduction**	Calmodulin [30,50], G-protein subunit β5 [93], KSR-2* [53,94], Rhophilin-2* [95], Trio* [52,96], TFG [54]

**Inflammation and Immunity**	Immunoglobulin lambda light chain [97], NFκB p105

**Cellular Proliferation**	AF-17 [98], RTEL-1* [99]

**Boldface font **indicates proteins identified as Pyk2-associated in multiple cell types

* indicates proteins identified with low abundance (< 3 spectral hits on a single peptide).

### Pyk2 F402Y is Defective in RhoA Activation

**Figure 5:**
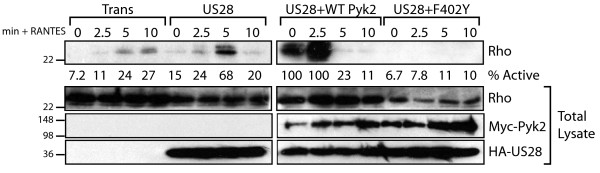
**Pyk2-F402Y Blocks pUS28 Signaling to RhoA**. FAK-/- fibroblasts were infected with Ad-Trans and Ad-pUS28 +/- Pyk2-WT or -F402Y for 18 hrs. Cells were stimulated with 40 ng/ml CCL5 for the indicated times. Lysates were immunoprecipitated with Rhotekin-RBD-GST Agarose and analyzed by western blot for RhoA. Input lysates were analyzed for total RhoA and to confirm adenovirus infection efficiency. The percent active RhoA was quantified via ImageJ densitometry of both IP and total lysate western blots.

We have previously demonstrated that activation of RhoA is necessary for pUS28-mediated SMC migration [16]. Therefore, we hypothesized that the dominant negative effect of the Pyk2 F402Y mutant is due to a defect in RhoA activation. To test this hypothesis, we performed a RhoA activation assay in cells expressing pUS28 that were stimulated with CCL5. For these experiments, Rhotekin-RhoA Binding Domain (RBD) conjugated beads were used to specifically pull-down active RhoA from FAK-/- cells expressing Trans only, or Trans + pUS28 with or without Pyk2-WT or Pyk2-F402Y. Percent of RhoA activated was calculated by comparing immunoprecipitated RhoA with total RhoA in the same sample. As expected, within 5 min, CCL5 stimulation of pUS28-expressing FAK-/- results in activation of 68% of total RhoA compared to a maximum of 27% activation in control Trans infected cells treated with CCL5 (Figure 5). Overexpression of Pyk2-WT markedly raised baseline activation of RhoA in pUS28 expressing cells and resulted in the activation of 100% of RhoA at 2.5 min post addition of CCL5. The phenomenon of increased baseline activation of Pyk2 and downstream effectors in the context of overexpression has been observed by others, and appears to be due to the Ca^2+^-mediated regulation of Pyk2 activity [30]. Conversely, overexpression of Pyk2-F402Y abrogated pUS28-mediated activation of RhoA, resulting in a maximum of 10% activation over the timecourse of stimulation (Figure 5). We observed some cellular toxicity in samples overexpressing Pyk2 F402Y which resulted in slightly lower total RhoA levels in these samples, but as each sample was internally controlled we believe this has no effect on the data as shown. These results demonstrate that activation of Pyk2 at Y402 is necessary for Pyk2-mediated signaling to RhoA and subsequent signaling leading to SMC migration.

### pUS28 Activates Pyk2 in Glioblastoma Cells

**Figure 6:**
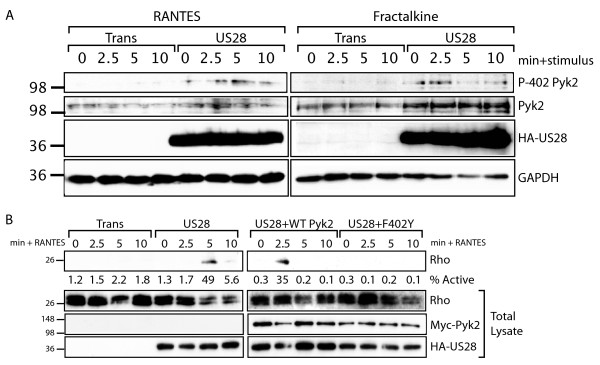
**pUS28 Activates Pyk2 and RhoA in U373 Glioblastoma Cells**. (A) U373 cells were infected with Ad-Trans or Ad-pUS28 for 18 hrs. Cells were stimulated with 40 ng/ml CCL5 (left) or 40 ng/ml CX3CL1 (right) for the indicated times and analyzed via western blot with a phospho-specific Pyk2-Y402 antibody or total Pyk2 antibody. (B) U373 were infected with Ad-Trans, Ad-pUS28 or Ad-pUS28 + Pyk2 WT or F402Y for 18 hrs. Cells were stimulated with 40 ng/ml CCL5 for the indicated times. Lysates were immunoprecipitated with Rhotekin-RBD-GST Agarose and analyzed by western blot for RhoA. Input lysates were analyzed for total RhoA and to confirm adenovirus infection efficiency. The percent active RhoA was quantified via ImageJ densitometry of both IP and total lysate western blots.

Having established a functional link between pUS28, Pyk2 and migration in SMC, we hypothesized that pUS28 mediates pro-migratory signaling to Pyk2 in other CMV-susceptible cell types. Although HCMV infection has been associated with poor clinical outcome in glioblastoma multiforme (GBM) patients, a clear mechanistic link between HCMV and GBM tumorigenesis has not been established [5,46,57]. However, many studies have associated aberrant activation of Pyk2 with increased invasiveness in GBM [41,58-60]. To test whether pUS28 can signal to Pyk2 in a glioma model, we examined phosphorylation of Pyk2 at the Y402 site in pUS28 adenovirus infected U373. Interestingly, we observed pUS28-specific phosphorylation of Pyk2 in U373 in response to both CCL5 and CX3CL1 stimulation (Figure 6A). Interestingly, Pyk2 phosphorylation in response to CX3CL1 stimulation is biphasic, with two separate peaks of phosphorlyation, the first at 2.5 and second at 10 min post-ligand addition. This biphasic pattern of phosphorylation at the Y402 site has been observed in other systems [37].

We tested the participation of Pyk2 in the pUS28-mediated activation of RhoA in this system. Similar to results obtained in FAK-/- fibroblasts, pUS28-specific activation of RhoA is readily observed in U373. pUS28-expressing U373 showed 49% activation of RhoA at 5 min post addition of CCL5. Adenovirus expression of WT Pyk2 resulted in similar activation of RhoA, but altered the timing of RhoA activation such that the peak of RhoA activation was seen at 2.5 min post addition of CCL5. Overexpression of the Pyk2 F402Y mutant prevents pUS28-mediated activation of RhoA resulting in less than 1% activation of RhoA throughout the timecourse of stimulation (Figure 6B).

In order to compare pUS28-mediated activation of Pyk2 in U373 to that seen in RSMC, we performed proteomics analysis of Pyk2 complexes in U373 (Figure 4 Table 2 and Additional File 2). The conditions for this experiment were the same as the previous experiment in RSMC. Overall, Pyk2 seems to have a greater number of binding partners in U373 underscoring the importance of Pyk2 in glioma cell biology. A total of 73 proteins were found associated with Pyk2 in this system, however only 18 of these were found specifically in pUS28-expressing samples (Figure 4A). In contrast to results seen in RSMC, nearly all of the proteins associated with Pyk2 in U373 were induced by signaling (Figure 4B), revealing low baseline activity of Pyk2 in these cells. Many of the same proteins binding to Pyk2 in response to pUS28 signaling were detected in samples from both cell types (Table 1 &2 , boldface text). However, there were a number of proteins that were unique to pUS28 signaling in U373. Importantly, several heat shock proteins of the Hsp70 family (BiP, mortalin and Hsc70) known to be involved in glioma tumorigenesis [61-64] were found to complex with Pyk2 in response to pUS28 signaling. Additionally, several regulators of the Wnt/beta-catenin/Tcf signaling pathway, also implicated in glioma pathogenesis [65,66] are associated with pUS28-activated Pyk2 complexes (Table 2).

**Table 2:** Pyk2 binding partners in pUS28-expressing U373

CATEGORY	PROTEIN(S) IDENTIFIED
**Cytoskeleton Structure and Dynamics**	β-actin, α1-actin, α2-actin, Nectin-3 [100], Myosin-9, Myosin light chain-6, Tropomyosin-α1 [72,90,91]

**Signal Transduction**	USP6 oncogene* [101], NIM1 kinase* [102], Copine-5* [103], Abl-interactor-2* [104], TFG [54]

**Inflammation and Immunity**	Immunoglobulin lambda light chain [97], NFκB p105

**Cellular Proliferation**	TCF-4 [50,65], AF-17 [98], RTEL-1* [99,105,106], N-acetyltransferase-10 [107], Integrator complex subunit 6 [108]

**Stress Response**	BiP [61,109], Mortalin [62,110], Hsc70 [63,64]

**Boldface font **indicates proteins identified as Pyk2 associated in multiple cell types

* indicates proteins identified with low abundance (< 3 spectral hits on a single peptide).

## Discussion

**Figure 7:**
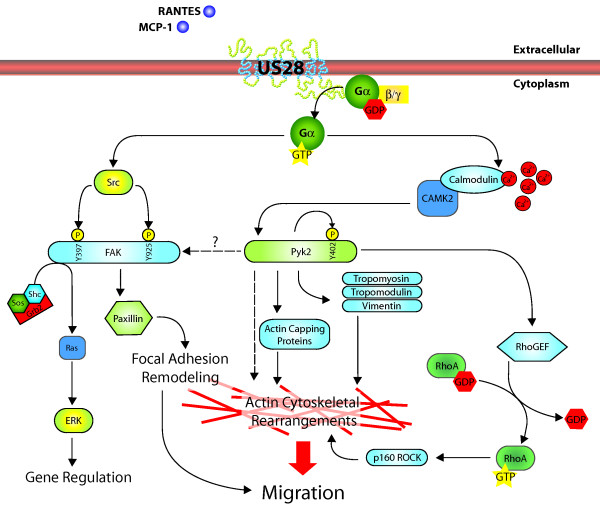
**pUS28-mediated Pro-migratory Signaling in SMC**. Schematic model for known components of US28-mediated pro-migratory signaling in response to CC-chemokine ligands.

In this paper, we examined the ability of the HCMV-encoded chemokine receptor pUS28 to activate Pyk2 and determined the role of Pyk2 in pUS28 mediated SMC migration (Figure 7). Pyk2 expression is limited to a subset of cell types *in vivo *including brain, hematopoetic cells, endothelial cells, SMC and fibroblasts. Interestingly, many of these cell types are capable of undergoing migration events in response to various external stimuli including integrin, growth factor, hormone and chemokine-mediated signals. There is very little consensus in the literature regarding the participation of Pyk2 in such migration events. Indeed, requirements for Pyk2 signaling, and downstream effectors of Pyk2 activation are highly cell type and signal type-specific [28].

### Pyk2 and migration in vascular smooth muscle

Migration of vascular SMC is induced by a variety of stimuli including growth factors, cytokines, extracellular matrix (ECM) components and peptide hormones. Pro-migratory signaling in SMC is best understood in the context of platelet-derived growth factor (PDGF) binding through receptor tyrosine kinases PDGFR-α and β. PDGF-mediated migration in SMC is characterized by Src-dependent activation of FAK, RhoA mediated activation of ROCK and subsequent phosphorylation and inhibition of myosin light chain phosphatase as well as activation of various other effector molecules via ERK, p38MAPK and JNK MAPK families [67]. Angiotensin II (Ang II) is another well-studied pro-migratory signal in SMC. Ang II binding to the GPCR angiotensin receptor type 1 (AT-1) initiates the only pro-migratory signaling cascade in SMC previously reported to involve Pyk2 [68]. In this signaling context, Pyk2 is activated by PKCδ [69] and signals to Rho/ROCK via association with PDZ-RhoGEF [26]. Additionally, Pyk2 has been identified as an essential signaling scaffold linking GPCR signaling to MAP kinase activation via Src activation upon binding to Tyr-402 autophosphorylated Pyk2 [31]. Activation of JNK and ERK MAP kinase pathways are necessary for Ang II-mediated SMC migration [26].

The results presented herein represent the first example of a chemokine-mediated pro-migratory signaling cascade in VSMC involving Pyk2. We have previously demonstrated that chemokine binding to the viral chemokine receptor pUS28 causes SMC migration via a Src-mediated activation of FAK and ERK [17], and the Gα12-mediated activation of RhoA and ROCK [16]. These results refine our understanding of the pUS28 receptor-proximal events leading to the activation of RhoA via the ligand-dependent phosphorylation of Pyk2 at Tyr-402. Pyk2 kinase activity is dispensable for pUS28-mediated migration in SMC, suggesting that Pyk2 participates primarily as a signaling scaffold in pUS28 pro-migratory signaling. We have previously demonstrated that pUS28-induced SMC migration is sensitive to the Src inhibitor PP-2 and that FAK functionally interacts with Src in pUS28-expressing cells stimulated with CCL5. We further demonstrated that Src activation is upstream of FAK in this pathway [17]. Similarly, pUS28 can signal to Pyk2 in FAK-/- fibroblasts suggesting that Pyk2 activation is upstream or independent of FAK in this signaling cascade. Our proteomics analysis of Pyk2 binding partners in the context of pUS28 signaling revealed several novel proteins that shed new insight into the function of Pyk2 in pro-migratory signaling. In particular, we show that Pyk2 interacts with several tropomyosin (TM) isoforms and the TM interacting protein tropomodulin isoform 3 (Tmod3) in response to pUS28 signaling. These proteins are well-characterized regulators of actin polymerization and cytoskeletal dynamics [70]. Tmod3 is broadly expressed in cells with dynamic actin filament structures and is unique among tropomodulin proteins in that, in addition to regulating the capping of actin filament pointed ends, it binds free actin monomers and may regulate the nucleation of new actin filaments [71]. Our discovery of an association of Pyk2 with TM isoforms is novel and particularly intriguing. TM proteins regulate the structure and specific properties of actin filaments but the mechanisms of TM regulation remain obscure [72]. One possible mechanism of TM regulation is phosphorylation. Indeed, the phosphorlyation of TM1 in response to ERK signaling in endothelial cells increases the formation of both actin stress fibers and focal adhesions contributing to increased cellular contractility [73]. Our results suggest that the Pyk2 signaling complex participates in cytoskeleton remodeling via modification of Tmod and TM proteins. Taken together, these results suggest that Pyk2 acts together with Src, FAK and other adaptor molecules to promote pro-migratory signaling and that Pyk2 phosphorylation at Tyr-402, is required for the downstream activation of RhoA. Our finding that the scaffolding activity of Pyk2 is separable from its kinase activity is novel in the literature. In the context of AngII-mediated SMC migration as well as migration of endothelial cells, Pyk2 kinase activity is an essential component of pro-migratory signaling [26,74].

### pUS28 signaling and CMV-associated malignancy

In this paper, we explore the contribution of pUS28 signaling to Pyk2 in migration processes associated with HCMV-mediated vascular disease. However, HCMV has been implicated in other disease processes in which the induction of cellular migration may play a role. In particular, HCMV infection has been associated with several human malignancies including prostate and colon cancers as well as glioblastoma multiforme (GBM) [75]. GBM is the most common and most malignant type of brain cancer and currently has no effective treatment [76]. HCMV DNA is present in over 90% of human malignant gliomas [1,77], suggesting a role for HCMV in glioma oncogenesis. A recent report has strengthened the connection between HCMV and GBM by demonstrating that vaccination against the HCMV tegument protein pp65 can improve survival in GBM patients [78]. However, the molecular mechanisms of HCMV participation in glioma development remain unclear.

pUS28 signaling has recently been reported to produce a transformed phenotype in transfected NIH-3T3 cells characterized by increased proliferative capacity, and increased expression of the pro-angiogenic marker VEGF. In addition, the study demonstrated that WT HCMV infection of a human glioblastoma cell line (U373) induces VEGF expression while a pUS28 deletion mutant of HCMV fails to induce VEGF upregulation in glioblastoma cells [10] implicating pUS28 signaling in glioma pathogenesis. However, this study did not explore the contribution of pUS28 ligand binding to the observed phenotypes.

### Pyk2 and glioma tumorigenesis

In addition to its role in vascular remodelling, Pyk2 has been identified as a pro-metastatic and pro-angiogenic molecule in several cancer models including small cell lung cancer [79,80], prostate cancer [81-83] and glioblastoma [41,58-60]. A number of elegant studies by Lipinski et. al. have established roles for Pyk2 and FAK in glioma tumorigenesis both *in vitro *and *in vivo*. In these studies, Pyk2 is demonstrated to promote migration and invasion of glioma cells while FAK controls their proliferation. The authors demonstrate that the migratory capacity of individual glioma cell lines can be correlated to the relative levels of Pyk2 expression and activation. Perhaps the most compelling evidence for the role of Pyk2 and FAK in glioma tumor progression utilized an *in vivo *xenograft model of glioblastoma in mice. In this study, Pyk2 deficient cells were observed to invade only locally and survival was significantly increased when mice were engrafted with Pyk2 or FAK deficient glioma cells compared to mice engrafted with wild-type glioma cells [60]. Our current study and previous work characterizing the pUS28-mediated activation of FAK suggest a mechanism for acceleration of glioma tumor formation by HCMV via pUS28-mediated signaling through the critical mediators of glioma tumor progression, Pyk2 and FAK. Our results are consistent with existing literature in which the Pyk2 FERM domain is established as a target for reducing glioma cell migration [58,84]. Pyk2 undergoes dimerization and autophosphorylation upon FERM domain interaction with Ca2+/calmodulin [30]. Therefore inhibition of the Pyk2 FERM domain by mutation or antibody targeting may prevent dimerization and phosphorylation of Pyk2 at Tyr-402 thereby preventing the activation of RhoA.

We observed the formation of a cell type specific Pyk2 complex in response to pUS28 signaling in U373 glioblastoma cells. This complex included several proteins previously implicated in glioma tumorigenesis, but never before identified as Pyk2 binding partners. Our data suggests at least two novel mechanisms for the participation of Pyk2 and pUS28 in glioma tumorigenesis. First, we identified TCF-4 and AF-17 binding to Pyk2 in response to pUS28 signaling. Both proteins are members of the Wnt/β-catenin signaling pathway that has recently been linked to glioma progression [65,66]. Indeed, siRNA mediated inhibition of this pathway decreases glioma proliferation and invasion capacity [65]. Interestingly, Pyk2 has been shown to phosphorylate β-catenin in endothelial cells [85] suggesting that Pyk2 may be involved in the activation of canonical β-catenin signaling in multiple cell types. Second, we discovered that pUS28 signaling caused Pyk2 to associate with three members of the Hsp70 family of molecular chaperone proteins. Interestingly, all three of these proteins are known to be specifically upregulated in glioma tissue and play various roles in the progression of glioma tumorigenesis [61-63] as well as other cancer models [86]. Mortalin, in particular, is functionally regulated via tyrosine phosphorylation [87]. Our results suggest that Pyk2, or kinases associated with Pyk2 may play a role in regulating the function of multiple Hsp70-family chaperones, thereby contributing to the proliferation and survival of tumor cells.

These findings refine our understanding of the pro-migratory signaling pathways activated by pUS28 in SMC and establish Pyk2 as an attractive target for the treatment of CMV-mediated vascular disease because it acts early in the pUS28 pro-migratory signaling cascade and its expression is restricted to a few cell types, reducing the opportunities for off-target effects. Further, these results suggest a role for HCMV pUS28 in pathologies associated with aberrant activation of Pyk2 including some malignancies.

## Conclusions

In this report, we determined the involvement of Pyk2 in pUS28 signaling and cellular migration (Figure 7). We found that Pyk2 autophosphorylation activity, but not kinase activity, is necessary for HCMV-mediated SMC migration. Furthermore, pUS28 signaling causes ligand-dependent phosphorylation of Pyk2 at Tyr-402 independent of pUS28 signaling to FAK. Pyk2 is incorporated into an active kinase complex in CCL5 stimulated SMC expressing pUS28. Pyk2 associates with a number of other proteins in response to pUS28 signaling including kinases, signaling molecules, cytoskeletal proteins, and molecular chaperones. We determined that although the Pyk2 autophosphorylation mutant (F402Y) does not prevent the formation of an activated kinase complex, it exerts a dominant negative effect on SMC migration by preventing the pUS28-mediated activation of the small G-protein RhoA. Importantly, the effect of pUS28 activating Pyk2 is not cell-type specific as we observed comparable activation of Pyk2 and RhoA via pUS28 signaling in U373 glioblastoma cells. This finding suggests that pUS28 signaling to Pyk2 may be important in glioma cell motility. In addition to establishing Pyk2 as a potential target in the treatment of HCMV-mediated vascular disease, and establishing a number of novel Pyk2-associated proteins, these results provide a potential link between pUS28 and CMV-associated malignancy.

## Competing interests

The authors declare that they have no competing interests.

## Authors' contributions

JEV participated in the design of the study, carried out all signaling and immunofluorescence assays, prepared samples for proteomics analysis and drafted the manuscript. SV coordinated the mass spectrometry and participated in MS data analysis. RM developed the RhoA activation assay. PS performed SMC migration assays and helped to edit the manuscript. LPT prepared samples for mass spectrometry and participated in data analysis, JIS participated in MS data analysis, DNS conceived of the study, participated in its design and coordination and helped to draft and edit the manuscript. All authors read and approved the final manuscript.

## Supplementary Material

Additional file 1 **Table S1: Mass Spectrometry Data for Pyk2 Complexes in RSMC**. For each Pyk2 associated protein, spectral hits are shown for each unique peptide over the timecourse of stimulation. Total spectral hits per peptide are shown to the right of the timecourse for each condition. Total peptides and spectral hits for each timepoint are shown below the list of peptides for each protein. Click here for file

Additional file 2 **Table S2: Mass Spectrometry Data for Pyk2 Complexes in U373**. For each Pyk2 associated protein, spectral hits are shown for each unique peptide over the timecourse of stimulation. Total spectral hits per peptide are shown to the right of the timecourse for each condition. Total peptides and spectral hits for each timepoint are shown below the list of peptides for each protein. Click here for file
