# Supplementary material for: HCMV pUS28 initiates pro-migratory signaling via activation of Pyk2 kinase
Source: Herpesviridae. 2010 Dec 7;1:2. doi: 10.1186/2042-4280-1-2 (PMC3050435; doi:10.1186/2042-4280-1-2)
Supplement: Additional file 1 — Table S1: Mass Spectrometry Data for Pyk2 Complexes in RSMC. For each Pyk2 associated protein, spectral hits are shown for each unique peptide over the timecourse of stimulation. Total spectral hits per peptide are shown to the right of the timecourse for each condition. Total peptides and spectral hits for each timepoint are shown below the list of peptides for each protein. [file 2042-4280-1-2-S1.PDF]

Additional File 1: Mass Spectrometry Data For Pyk2 Complexes in RSMC

For each Pyk2 associated protein, spectral hits are shown for each unique peptide over the timecourse of stimulation. Total spectral hits per peptide are shown to the right of the timecourse for each condition. Total peptides and spectral hits for each timepoint are shownbelow the list of peptides for each protein

	Condition	Pyk2						Pyk2 Total	Pyk2&US28						Pyk2&US28 Total
Description	PeptideSequence	0	5	10	15	30	60		0	5	10	15	30	60	Min post-stimulation
MYH9 Isoform 1 of Myosin-9	K.AKQTLENER.G										1	1			2
	K.AKQTLENERGELANEVK.V								1	1	3				5
	K.ALELDSNLYR.I									1	1			1	3
	K.ANLQIDQINTDLNLER.S	1	1		2		1	5	2	2	3	1	1	2	11
	K.DDVGKSVHELEK.S									1	1				2
	K.DFSALESQLQDTQELLQEENR.Q	4	2	1	2		1	10	2	6	7	6	5	6	32
	K.DFSALESQLQDTQELLQEENRQK.L	2		2	2			6	2	2	2			2	8
	K.DFSALESQLQDTQELLQEENRQKLSLSTK.L			3	2			5	2					2	4
	K.DLEGLSQR.H										4				4
	K.FDQLLAEEK.T						1	1	1	1	1		1	1	5
	K.FDQLLAEEKTISAK.Y													1	1
	K.GMFRTVGQLYKEQLAKLMATLRNTNPNFVR.C										1				1
	K.HEAMITDLEER.L										2		1		3
	K.HSQAVEELAEQLEQTKR.V	1					1	2	2	2	3	1		2	10
	K.IAQLEEQLDNETK.E		1					1			3	2	1		6
	K.IAQLEEQLDNETKER.Q								1	1	2			1	5
	K.KANLQIDQINTDLNLER.S	1			1			2	2	2	1		2	1	8
	K.KDQGELERQLLQANPILEAFGNAK.T													1	1
	K.KEEELQAALAR.V												1		1
	K.KFDQLLAEEK.T						2	2	2		1	1	1	1	6
	K.KFDQLLAEEKTISAK.Y	2		2	2		1	7	3					2	5
	K.KKMQQNIQELEEQLEEEESAR.Q										1				1
	K.KMQQNIQELEEQLEEEESAR.Q									1	1				2
	K.KQKKFDQLLAEEKTISAK.Y						1	1						2	2
	K.KRHEMPPHIYAITDTAYR.S	2		2	2		1	7	2	1	3			2	8
	K.KVEAQLQELQVK.F	1						1	1	1	2	2	1	1	8
	K.LDPHLVLDQLR.C									1	1				2
	K.LKNKHEAMITDLEER.L	1		1				2		4	4	2		2	12
	K.LKSMEAEMIQLQEELAAAER.A									1	1				2
	K.LQEMEGTVKSK.Y													1	1
	K.LQKDLEGLSQR.H									1	2	1		1	5
	K.LQLEKVTTEAK.L						1	1							
	K.LRLEVNLQAMK.A										1				1
	K.LTKDFSALESQLQDTQELLQEENR.Q	2		1	1		2	6	2	2	4	2		2	12
	K.LTKDFSALESQLQDTQELLQEENRQK.L	2		2	2		1	7	2	2	2			2	8
	K.LTKDFSALESQLQDTQELLQEENRQKLSLSTK.L				2			2	1					2	3
	K.MQQNIQELEEQLEEEESAR.Q	2	2				1	5		4	4	1	2	2	13
	K.MQQNIQELEEQLEEEESARQKLQLEK.V													3	3
	K.NFINNPLAQADWAAK.K		2					2		1	1	1	1	1	5
	K.NKHEAMITDLEER.L									3	4	2	1	3	13
	K.NMDPLNDNIATLLHQSSDKFVSELWK.D										2				2
	K.NMDPLNDNIATLLHQSSDKFVSELWKDVDR.I									3	2			1	6
	K.QKKFDQLLAEEKTISAK.Y	1		1				2							
	K.QKRDLGEELEALKTELEDTLDSTAAQQELR.S			1				1						3	3
	K.QTLENERGELANEVK.V									2	2	2			6
	K.RALEQQVEEMKTQLEELEDELQATEDAK.L										2				2
	K.RDLGEELEALKTELEDTLDSTAAQQELR.S									1	2				3
	K.RHEMPPHIYAITDTAYR.S										1			1	2
	K.RQAQQERDELADEIANSSGK.G									1	1				2
	K.RQLEEAEEEAQR.A									2	3	2	2		9
	K.RQLEEAEEEAQRANASR.R										1				1
	K.SKKDQGELER.Q										1				1
	K.SKKDQGELERQLLQANPILEAFGNAK.T			2	2			4						2	2
	K.SMEAEMIQLQEELAAAER.A	2					1	3	2	4	5	2	4	4	21
	K.SSKLTKDFSALESQLQDTQELLQEENR.Q													1	1
	K.SSKLTKDFSALESQLQDTQELLQEENRQK.L			1	2			3						1	1
	K.SVHELEKSKR.A								1					1	2
	K.TDLLLEPYNK.Y										1	1	1	1	4
	K.THEAQIQEMR.Q												2		2
	K.TQLEELEDELQATEDAK.L									1	3		3		7
	K.TQLEELEDELQATEDAKLR.L									1	3				4
	K.VAAYDKLEK.T										1				1
	K.VEAQLQELQVK.F	1					1	2	1	1	1	1	1	1	6
	K.VKVNKDDIQK.M						3	3	2	1		1		1	5
	K.VSHLLGINVTDFTR.G	1	2	1	1		1	6	2	2	3	2	1	2	12
	K.YLYVDKNFINNPLAQADWAAK.K		1					1		2	2	1	2	1	8
	R.ALEEAMEQK.A										4				4
	R.ALEEAMEQKAELER.L	1						1	1	1	2			1	5
	R.ALEEAMEQKAELERLNK.Q								1					2	3
	R.ALEEAMEQKAELERLNKQFR.T													1	1
	R.ALEQQVEEMK.T									1	1	1	1		4
	R.ALEQQVEEMKTQLEELEDELQATEDAK.L	2	1					3		3	8	3	2	2	18
	R.ALEQQVEEMKTQLEELEDELQATEDAKLR.L								1	2	3				6
	R.ALEQQVEEMKTQLEELEDELQATEDAKLRLEVNLQAMK.A									2	3				5
	R.ASREEILAQAKENEK.K	1						1		1	2	1		1	5
	R.ASREEILAQAKENEKK.L									1	1				2
	R.ASREEILAQAKENEKKLK.S													1	1
	R.DELADEIANSSGK.G										1		2		3
	R.DLGEELEALKTELEDTLD.S										1				1
	R.DLGEELEALKTELEDTLDSTAAQQELR.S	5	3	4	2		3	17	4	8	7	4	5	6	34
	R.DLGEELEALKTELEDTLDSTAAQQELRSK.R								1					1	2
	R.DLGEELEALKTELEDTLDSTAAQQELRSKR.E								1					2	3
	R.DLQGRDEQSEEK.K									1	1				2
	R.DLQGRDEQSEEKKK.Q										1				1
	R.EEILAQAKENEK.K										1				1
	R.ELEDATETADAMNR.E	1	1				1	3	1	3	4		3	3	14
	R.ELEDATETADAMNREVSSLKNKLR.R			2	1			3							
	R.EMEAELEDER.K									1	1	1	1		4
	R.EMEAELEDERK.Q								1	1	1	1	1		5
	R.EVSSLKNKLR.R													1	1
	R.FLSNGHVTIPGQQDKDMFQETMEAMR.I			1				1		2	3			2	7
	R.HEMPPHIYAITDTAYR.S									2	2			1	5
	R.IAQLEEELEEEQGNTELINDR.L	2					2	4	2	3	4	2	2	2	15
	R.IAQLEEELEEEQGNTELINDRLK.K	2		1			2	5	2					2	4
	R.IAQLEEELEEEQGNTELINDRLKK.A								2					1	3
	R.IIGLDQVAGMSETALPGAFK.T									3	5	3	2	4	17
	R.IKVGRDYVQK.A													2	2
	R.INFDVNGYIVGANIETYLLEK.S									4	4				8
	R.KKVEAQLQELQVK.F						1	1	2	2	4	2	2	4	16
	R.KLQRELEDATETADAMNR.E								1						1
	R.LEVNLQAMK.A								1		2	1		1	5
	R.LQQELDDLLVDLDHQR.Q	1			2		2	5	2	3	3	2	2	2	14

Additional File 1: Mass Spectrometry Data For Pyk2 Complexes in RSMC

	Condition	Pyk2						Pyk2 Total	Pyk2&US28						Pyk2&US28 Total
**Description**	**PeptideSequence**	0	5	10	15	30	60		0	5	10	15	30	60	**Min post-stimulation**
	R.NTDQASMPDNTAAQK.V	2	1				1	4	1	1	2	1	2	1	8
	R.NTDQASMPDNTAAQKVSHLLGINVTDFTR.G	2		2	2		1	7	3					2	5
	R.QAQQERDELADEIANSSGK.G									2	2	1			5
	R.QAQQERDELADEIANSSGKGALALEEK.R													1	1
	R.QAQQERDELADEIANSSGKGALALEEKR.R													1	1
	R.QKHSQAVEELAEQLEQTK.R										1				1
	R.QKHSQAVEELAEQLEQTKR.V									1				1	2
	R.QLEEAEEEAQR.A	1	1					2	1	2	3	1	1	1	9
	R.SMMQDREDQSILCTGESGAGKTENTKK.V													2	2
	R.TEMEDLMSSK.D									1	1	1	1	1	5
	R.TEMEDLMSSKDDVGK.S									1	3	1		1	6
	R.TEMEDLMSSKDDVGKSVHELEK.S								1						1
	R.TFHIFYYLLSGAGEHLK.T										2				2
	R.TFHIFYYLLSGAGEHLKTDLLLEPYNKYR.F										3				3
	R.TVGQLYKEQLAK.L			1			2	3		1	1	1		3	6
	R.TVGQLYKEQLAKLMATLRNTNPNFVR.C										2				2
	R.VISGVLQLGNIVFK.K										2			1	3
	R.VISGVLQLGNIVFKK.E			1	1			2	1	2	2	2		1	8
	R.VVFQEFR.Q									2					2
**MYH9 Isoform 1 of Myosin-9 Totals: Unique Peptides**	**121**	46	18	32	31		35	162	66	119	196	64	61	125	631
MYH10 Isoform 1 of Myosin-10	K.AMVNKDDIQK.M									1	1				2
	K.IGQLEEQLEQEAK.E										1				1
	K.KEEELQGALAR.G										1		2		3
	K.KLDAQVQELHAK.V								1		1			1	3
	K.KLVWIPSER.H								1	1	1			1	4
	K.KQELEEILHDLESR.V									2	1				3
	K.LKSLEAEILQLQEELASSER.A										1				1
	K.LQNELDNVSTLLEEAEK.K										3		1		4
	K.LQNELDNVSTLLEEAEKK.G										2				2
	K.LVQEQGSHSK.F											1			1
	K.MQAHIQDLEEQLDEEEGAR.Q									1	3				4
	K.NILAEQLQAETELFAEAEEMR.A									2	4	1	1		8
	K.QVLALQSQLADTK.K									1	1	1	1	1	5
	K.RQLEEAEEEATR.A										1				1
	K.SALLDEKR.R								1						1
	K.SLEAEILQLQEELASSER.A	1						1		4	3	1	4	4	16
	K.VIQYLAHVASSHK.G				1			1		1	2			1	4
	R.AAANKLVR.R										1				1
	R.ADMEDLMSSKDDVGKNVHELEK.S										2				2
	R.ALEEALEAKEEFER.Q									1	1				2
	R.ALEQQVEEMR.T									1	1	1	1		4
	R.AVIYNPATQADWTAK.K								1	1	1		2	1	6
	R.DLQTRDEQNEEK.K									1					1
	R.DLSEELEALKTELEDTLDTTAAQQELR.T	1						1	3	2	4	1	3	2	15
	R.ELDDATEANEGLSR.E	1					1	2	1	1	1	1	1	1	6
	R.ELEAELEDER.K										1				1
	R.ELEAELEDERK.Q										2				2
	R.ELQAQIAELQEDFESEK.A									3	2	2	1		8
	R.GDDETLHK.N										1				1
	R.GDDETLHKNNALK.V										1				1
	R.GGPISFSSSR.S									1	1		1	1	4
	R.HADQYKEQMEK.A										1				1
	R.HATALEELSEQLEQAK.R										1				1
	R.HATALEELSEQLEQAKR.F						1	1	2	1	2			1	6
	R.HGFEAASIKEER.G										1				1
	R.IAQLEEELEEEQSNMELLNDR.F									1	2		1		4
	R.IAQLEEELEEEQSNMELLNDRFR.K													1	1
	R.KKLDAQVQELHAK.V										2				2
	R.LEVNMQAMK.A										1				1
	R.LQQELDDLTVDLDHQR.Q										1				1
	R.NKQEVMISDLEER.L										1				1
	R.NTDQASMPENTVAQK.L										1				1
	R.QLEEAEEEATR.A								1	1	1	1	1		5
	R.QLLQANPILESFGNAK.T									1	2			2	5
	R.RGGPISFSSSR.S	1						1	1		1		1	1	4
	R.TGLEDPERYLFVDR.A													1	1
	Y.LAHVASSHK.G										1				1
**MYH10 Isoform 1 of Myosin-10 Totals: Unique Peptides:**	**47**	4			1		2	7	12	28	63	10	21	19	153
ACTB Actin, cytoplasmic 1	A.PEEHPVLLTEAPLNPK.A			1				1							
	E.TFNTPAMYVAIQAVLSLYASGR.T	1						1		1	2		1	4	8
	K.DLYANTVLSGGTTMYPGIADR.M	3	1	3				7	2	4	5		2	3	16
	K.LCYVALDFEQEMATAASSSSLEK.S			2				2						5	5
	K.MTQIMFETFNTPAMYVAIQAVLSLYASGR.T									2	3				5
	K.QEYDESGPSIVHR.K									1			1	1	3
	K.YSVWIGGSILASLSTFQQMWISKQEYDESGPSIVHRK.C										1				1
	R.DIKEKLCYVALDFEQEMATAASSSSLEK.S			3				3						2	2
	R.EIVRDIKEKLCYVALDFEQEMATAASSSSLEK.S			2				2						2	2
	R.FRCPEALFQPSFLGMESCGIHETTFNSIMK.C										1			2	3
	R.GYSFTTTAER.E	1	1		1		1	4	1	2	2	1	1	1	8
	R.KDLYANTVLSGGTTMYPGIADR.M										1			1	2
	R.TTGIVMDSGDGVTHTVPIYEGYALPHAILR.L	3	1	3	2		2	11	2	3	4	1		1	11
	R.VAPEEHPVLLTEAPLNPK.A	2	2	3	2	1	5	15	3	2	2	3	2	2	14
	R.VAPEEHPVLLTEAPLNPKANR.E								1					1	2
**ACTB Actin, cytoplasmic 1 Totals: Unique Peptides**	**15**	10	5	17	5	1	8	46	9	15	21	5	7	25	82
MYL6B;MYL6 Isoform Non-muscle of Myosin light polypeptide 6	K.DQGTYEDYVEGLR.V								1	1	1		1	1	5
	K.NKDQGTYEDYVEGLR.V	1		2	1			4	4	4	5	3	3	4	23
	K.NKDQGTYEDYVEGLRVFDKEGNGTVMGAEIR.H								1					2	3
	K.VLDFEHFLPMLQTVAK.N	2	1	5	1			9	4	6	8	3	3	4	28
	K.VLGNPKSDEMNVK.V			1	1			2	1					2	3
	K.VLGNPKSDEMNVKVLDFEHFLPMLQTVAK.N			3				3	3					2	5
	R.ALGQNPTNAEVLKVLGNPKSDEMNVK.V			2	2			4	2					1	3
	R.TGDGKILYSQCGDVMR.A				1			1						3	3
**MYL6B;MYL6 Isoform Non-muscle of Myosin light polypeptide 6 Totals: Unique Peptides**	**8**	3	1	13	6			23	16	11	14	6	7	19	73
TPM1 tropomyosin 1 alpha chain isoform 2	K.AEADVASLNR.R										2	1		1	4
	K.EENLSMHQMLDQTLLELNNM.-										2				2
	K.HIAEDADR.K									1	1		1		3

Additional File 1: Mass Spectrometry Data For Pyk2 Complexes in RSMC

	Condition	Pyk2						Pyk2 Total	Pyk2&US28						Pyk2&US28 Total
**Description**	**PeptideSequence**	0	5	10	15	30	60		0	5	10	15	30	60	**Min post-stimulation**
	K.HIAEDADRKYEEVAR.K								2					1	3
	K.LDKENALDRAEQAEADKKAAEDR.S									1	1			1	3
	K.LEEAEKAADESER.G		1					1	3	1	3	1	3		11
	K.LVIIESDLER.A													1	1
	K.SIDDLEEK.V										1	1	1		3
	K.SIDDLEEKVAHAKEENLSMHQMLDQTLLELNNM.-								2						2
	K.VAHAKEENLSMHQMLDQTLLELNNM.-									1	3				4
	K.VLSDKLKEAETR.A										2				2
	R.AQKDEEKMEIQEIQLK.E								1					1	2
	R.AQKDEEKMEIQEIQLKEAK.H								1					2	3
	R.GMKVIESR.A								1						1
	R.IQLVEEELDR.A	1	1					2		2	1	1	2	1	7
	R.IQLVEEELDRAQER.L								2	2	1	2		2	9
	R.IQLVEEELDRAQERLATALQKLEEAEK.A													1	1
	R.IQLVEEELDRAQERLATALQKLEEAEKAADESER.G								1					1	2
	R.KLVIIESDLER.A										1	1	1	1	4
	R.KLVIIESDLERAEER.A									1					1
	R.KLVIIESDLERAEERAELSEGK.C								1	1				1	3
	R.LATALQKLEEAEK.A	1						1		2	1			2	5
	R.LATALQKLEEAEKAADESER.G	1		2			2	5	2	2	1			4	9
	R.LATALQKLEEAEKAADESERGMK.V								2					2	4
	R.RIQLVEEELDR.A		1					1		1	1	1	2	2	7
	R.RIQLVEEELDRAQER.L	2		2				4	1	2	1			2	6
**TPM1 tropomyosin 1 alpha chain isoform 2 Totals: Unique Peptides**	**26**	5	3	4			2	14	19	17	22	8	10	26	102
TPM2 Isoform 3 of Tropomyosin beta chain	K.EENVEIHQTLDQTLLELNNL.-										3		1	2	6
	K.LEKTIDDLEETLASAKEENVEIHQTLDQTLLELNNL.-													1	1
	K.TIDDLEETLASAK.E										1		2		3
	K.TIDDLEETLASAKEENVEIHQTLDQTLLELNNL.-	2	2	3			1	8	2	3	5	3	3	3	19
	R.ARQLEEELR.T								2		2				4
	R.KLVILEGELERSEERAEVAESR.A								1						1
	R.SEERAEVAESR.A									1					1
**TPM2 Isoform 3 of Tropomyosin beta chain Totals: Unique Peptides**	**7**	2	2	3			1	8	5	4	11	3	6	6	35
ACTA1 Actin, alpha skeletal muscle	K.DLYANNVMSGGTTMYPGIADR.M	1						1			1		3		4
	K.IWHHTFYNELR.V	1	1	1				3	1	2	1	2	1	1	8
	K.YPIEHGIITNWDDMEKIWHHTFYNELR.V	2						2	6	2	3			3	14
	R.KDLYANNVMSGGTTMYPGIADR.M									1					1
**ACTA1 Actin, alpha skeletal muscle Totals: Unique Peptides**	**4**	4	1	1				6	7	5	5	2	4	4	27
VIM Vimentin	D.FSLADAINTEFK.N										1				1
	K.FADLSEAANR.N	1						1	1		1				2
	K.ILLAELEQLK.G										1				1
	K.ILLAELEQLKGQGK.S								1		2				3
	K.LQEEMLQR.E										1				1
	K.VELQELNDR.F										1				1
	R.DGQVINETSQHHDDLE.-						2	2	1						1
	R.EKLQEEMLQR.E										1				1
	R.FLEQQNKILLAELEQLKGQGK.S			2				2	2						2
	R.ISLPLPNFSSLNLR.E										2				2
	R.KVESLQEEIAFLK.K										1				1
	R.LGDLYEEEMR.E										1				1
	R.LLQDSVDFSLADAINTEFK.N			1				1	1	1	2				4
	R.LLQDSVDFSLADAINTEFKNTR.T			2				2							
	R.QDVDNASLAR.L									1	2				3
	R.TNEKVELQELNDRFANYIDKVR.F			2				2	2						2
**VIM Vimentin Totals: Unique Peptides**	**16**	1		7			2	10	8	2	16				26
TMOD3 Tropomodulin-3	K.MLEENTNILK.F										1				1
	K.QLETVLDDLDPENALLPAGFR.Q								1	2	5	1	2		11
	R.AANAITKNNDLVR.K			1			1	2	1					2	3
**TMOD3 Tropomodulin-3 Totals: Unique Peptides**	**3**			1			1	2	2	2	6	1	2	2	15
TPM1 Isoform 1 of Tropomyosin alpha-1 chain	K.GTEDELDKYSEALKDAQEKLELAEK.K												2		2
	K.LKGTEDELDKYSEALKDAQEK.L												1		1
	K.LKGTEDELDKYSEALKDAQEKLELAEK.K									1	2		1		4
	K.QLEDELVSLQK.K									1	1	1	2	1	6
	R.SKQLEDELVSLQK.K									1	2	1	1	1	6
	R.SKQLEDELVSLQKK.L													1	1
**TPM1 Isoform 1 of Tropomyosin alpha-1 chain Totals: Unique Peptides**	**6**									3	5	2	7	3	20
TPM4 Isoform 1 of Tropomyosin alpha-4 chain	K.HIAEEADR.K									2			1		3
	K.HIAEEADRKYEEVAR.K								1	1				2	4
	K.LVILEGELER.A									1			1		2
	K.TIDDLEEKLAQAK.E											1			1
	K.TIDDLEEKLAQAKEENVGLHQTLDQTLNELNCI.-			2				2						3	3
	R.KLVILEGELER.A									1	2		1	1	5
**TPM4 Isoform 1 of Tropomyosin alpha-4 chain Totals: Unique Peptides**	**6**			2				2	1	5	2	1	3	6	18
GSN Isoform 1 of Gelsolin	K.DSQEEEKTEALTSAK.R									1	2		2		5
	K.QGFEPPSFVGWFLGWDDDYWSVDPLDR.A								1		1				2
	K.QTQVSVLPEGGETPLFKQFFK.N			1				1							
	M.VVEHPEFLK.A	1						1							
	R.QGQIIYNWQGAQSTQDEVAASAILTAQLDEELGGTPVQSR.V									2	1			2	5
	R.YIETDPANR.D												1		1
**GSN Isoform 1 of Gelsolin Totals: Unique Peptides**	**6**	1		1				2	1	3	4		3	2	13
GNB5 Isoform 1 of Guanine nucleotide-binding protein subunit beta-5	I.IFGASSVDFSLSGRLLFAG.Y	1		1				2		2	2	2	2	1	9
**GNB5 Isoform 1 of Guanine nucleotide-binding protein subunit beta-5 Totals: Unique Peptides**	**1**	1		1				2		2	2	2	2	1	9
MYH11 Myosin-11	K.DDVGKNVHELEK.S										1				1
	R.DLGEELEALKTELEDTLDSTATQQELR.A	2	1	1				4			2				2
	R.ELDEATESNEAMGR.E						1	1							
	R.INFDVTGYIVGANIETYLLEK.S										1				1

Additional File 1: Mass Spectrometry Data For Pyk2 Complexes in RSMC

	**Condition**	**Pyk2**						Pyk2 Total	**Pyk2&US28**						Pyk2&US28 Total
**Description**	**PeptideSequence**	0	5	10	15	30	60		0	5	10	15	30	60	**Min post-stimulation**
**MYH11 Myosin-11 Totals: Unique Peptides**	**4**	2	1	1			1	5			4				4
TPM2 Isoform 1 of Tropomyosin beta chain	K.ATDAEADVASLNR.R	1	2				1	4		1	1	2	1	1	6
	K.HIAEDSDR.K									1	2		3		6
	K.HIAEDSDRKYEEVAR.K								1					1	2
	K.KATDAEADVASLNR.R		1					1	1	1	1	1	2	2	8
	K.KATDAEADVASLNRR.I													1	1
**TPM2 Isoform 1 of Tropomyosin beta chain Totals: Unique Peptides**	**5**	1	3				1	5	2	3	4	3	6	5	23
TPM3 Isoform 2 of Tropomyosin alpha-3 chain	K.IQVLQQQADDAEER.A									1	1		1	1	4
	K.TIDDLEDKLK.C										1			1	2
	R.KIQVLQQQADDAEER.A									2	2		2	1	7
	R.MLDQTLLDLNEM.-								1					1	2
**TPM3 Isoform 2 of Tropomyosin alpha-3 chain Totals: Unique Peptides**	**4**								1	3	4		3	4	15
IGL@ IGL@ protein	K.ADSSPVKAGVETTTPSK.Q								1						1
	K.AGVETTTPSK.Q								1			1	1		3
	K.QSNNKYAASSYLSLTPEQWK.S								1						1
	K.YAASSYLSLTPEQWK.S									2	1	1	2		6
**IGL@ IGL@ protein Totals: Unique Peptides**	**4**								3	2	1	2	3		11
TUBB2C Tubulin beta-2C chain	K.GHYTEGAELVDSVLDVVR.K										1				1
	K.GHYTEGAELVDSVLDVVRK.E										1				1
	R.LHFFMPGFAPLTSR.G										1				1
	R.SGPFGQIFRPDNFVFGQSGAGNNWAK.G										3				3
**TUBB2C Tubulin beta-2C chain Totals: Unique Peptides**	**4**										6				6
CALM1;CALM3;CALM2 Calmodulin	K.MKDTDSEEEIR.E												1		1
	R.SLGQNPTEAELQDMINEVDADGNGTIDFPEFLTMMAR.K									2			2		4
**CALM1;CALM3;CALM2 Calmodulin Totals: Unique Peptides**	**2**									2			3		5
CAPZA1 F-actin-capping protein subunit alpha-1	K.FITHAPPGEFNEVFNDVR.L								1	1	2			2	6
**CAPZA1 F-actin-capping protein subunit alpha-1 Totals: Unique Peptides**	**1**								1	1	2			2	6
TFG Protein TFG	R.NRPPFGQGYTQPGPGYR.-	1		1				2	3	2			1	1	7
**TFG Protein TFG Totals: Unique Peptides**	**1**	1		1				2	3	2			1	1	7
MLLT6 Protein AF-17	G.SMGGGGSGFISGRRSR.S		1					1		1	1			1	3
**MLLT6 Protein AF-17 Totals: Unique Peptides**	**1**		1					1		1	1			1	3
TPM3 tropomyosin 3 isoform 1	K.YSQKEDKYEEEIK.I												1		1
	R.ALKDEEKMELQEIQLKEAK.H								1					1	2
	R.KLVIIEGDLER.T									2			1		3
**TPM3 tropomyosin 3 isoform 1 Totals: Unique Peptides**	**3**								1	2			2	1	6
CAPZB Isoform 1 of F-actin-capping protein subunit beta	K.TKDIVNGLR.S										1			1	2
	R.LVEDMENK.I										1				1
	R.LVEDMENKIR.S													1	1
	R.RLPPQQIEK.N										1				1
	R.STLNEIYFGK.T										1				1
**CAPZB Isoform 1 of F-actin-capping protein subunit beta Totals: Unique Peptides**	**5**										4			2	6
KSR2 Isoform 1 of Kinase suppressor of Ras 2	R.KKNKLKPPGTPPPSSRKL.I												1		1
	R.NAELDGFPQ.L										1				1
**KSR2 Isoform 1 of Kinase suppressor of Ras 2 Totals: Unique Peptides**	**2**										1		1		2
TRIO Isoform 3 of Triple functional domain protein	G.HTSAVIVENPDGTLKK.S										1				1
**TRIO Isoform 3 of Triple functional domain protein Totals: Unique Peptides**	**1**										1				1
CAPZA2 F-actin-capping protein subunit alpha-2	K.FIIHAPPGEFNEVFNDVR.L										2				2
**CAPZA2 F-actin-capping protein subunit alpha-2 Totals: Unique Peptides**	**1**										2				2
RTEL1 Isoform 4 of Regulator of telomere elongation helicase 1	R.AQPVLDPTGNFPDALDQLCGST.S								1		1				2
**RTEL1 Isoform 4 of Regulator of telomere elongation helicase 1 Totals: Unique Peptides**	**1**								1		1				2
RHPN2 Rhophilin-2	K.KISKKLSFLSWGTN.K										1				1
**RHPN2 Rhophilin-2 Totals: Unique Peptides**	**1**										1				1
NFKB1 Isoform 2 of Nuclear factor NF-kappa-B p105 subunit	R.RLEPVVSDAIYDSK.A								1		1		1		3
**NFKB1 Isoform 2 of Nuclear factor NF-kappa-B p105 subunit Totals: Unique Peptides**	**1**								1		1		1		3
