# Supplementary material for: HCMV pUS28 initiates pro-migratory signaling via activation of Pyk2 kinase
Source: Herpesviridae. 2010 Dec 7;1:2. doi: 10.1186/2042-4280-1-2 (PMC3050435; doi:10.1186/2042-4280-1-2)
Supplement: Additional file 2 — Table S2: Mass Spectrometry Data for Pyk2 Complexes in U373. For each Pyk2 associated protein, spectral hits are shown for each unique peptide over the timecourse of stimulation. Total spectral hits per peptide are shown to the right of the timecourse for each condition. Total peptides and spectral hits for each timepoint are shown below the list of peptides for each protein. [file 2042-4280-1-2-S2.PDF]

Additional File 2: Mass Spectrometry Data for Pyk2 Complexes in U373

For each Pyk2 associated protein, spectral hits are shown for each unique peptide over the timecourse of stimulation. Total spectral hits per peptide are shown to the right of the timecourse for each condition. Total peptides and spectral hits for each timepoint are shownbelow the list of peptides for each protein

	Condition	Pyk2						Pyk2 Total	US28&Pyk2						US28&Pyk2 Total
Description	PeptideSequence	0	5	10	15	30	60		0	5	10	15	30	60	Min post-stimulation
TFG Protein TFG	K.AQLGEDIRR.I								1			1			2
	K.LLSNDEVTIK.Y					1		1	1	1			1		3
	K.NVMSAFGLTDDQVSGPPSAPAEDR.S					3	2	5					2		2
	R.GKLLSNDEVTIK.Y	1			1	1		3		1	1	1	1		4
	R.NRPPFGQGYTQPGPGYR.-	4	2	2	5	1	1	15	3	3	4	4	3		17
	R.RIPIHNEDITYDELVLMMQR.V		2			3	3	8	1	1			1		3
TFG Protein TFG Totals: Unique Peptides	6	5	4	2	6	9	6	32	6	6	5	6	8		31
IGL@ IGL@ protein	K.AAPSVTLFPPSSEELQANK.A					1		1	1	2			1		4
	K.AAPSVTLFPPSSEELQANKATLVCLISDFYPGAVTVAWK.A											2			2
	K.ADSSPVKAGVETTTPSK.Q	1		1		1		3	1						1
	K.AGVETTKPSK.Q								1	1					2
	K.AGVETTTPSK.Q			2		1	1	4	1	2	1		1		5
	K.QSNNKYAASSYLSLTPEQWK.S	3		1	1			5				1			1
	K.VTVLGQPK.A		1	1		1		3							
	K.YAASSYLSLTPEQWK.S	1	1	2	1	2	2	9	2	2	1	2	2		9
	R.SYSCQVTHEGSTVEK.T											2			2
IGL@ IGL@ protein Totals: Unique Peptides	9	5	2	7	2	6	3	25	6	7	2	7	4		26
HSPA9 Stress-70 protein, mitochondrial	K.DAGQISGLNVLR.V	1						1		1	1	1	1		4
	K.NAVITVPAYFNDSQR.Q	1						1				1			1
	K.VQQTVQDLFGR.A												1		1
	R.AQFEGIVTDLIR.R					1	1	2					1		1
	R.KDSETGENIR.Q									1					1
	R.QAASSLQQASLK.L								1						1
	R.QAVTNPNNTFYATK.R											1			1
	R.TTPSVVAFTADGER.L	1				1	1	3				1			1
HSPA9 Stress-70 protein, mitochondrial Totals: Unique Peptides	8	3				2	2	7	1	2	1	4	3		11
ACTA2 Actin, aortic smooth muscle	K.AGFAGDDAPR.A					1	1	2	2	1	1	1	1		6
	K.DLYANNVLSGGTTMYPGIADR.M								4						4
	K.DSYVGDEAQSK.R								1						1
	K.DSYVGDEAQSKR.G								1						1
	K.EITALAPSTMK.I					1		1	2			1	1		4
	K.IWHHSFYNELR.V								2						2
	K.QEYDEAGPSIVHR.K								1						1
	K.YPIEHGIITNWDDMEK.I								3						3
	R.AVFPSIVGRPR.H	1	1		1	1	1	5	2	1	1	1	1		6
	R.GYSFVTTAER.E								2						2
	R.KDLYANNVLSGGTTMYPGIADR.M								2						2
	R.MQKEITALAPSTMK.I								1		1	2			4
	R.TTGIVLDSGDGVTHNVPIYEGYALPHAIMR.L								1						1
	R.VAPEEHPTLLTEAPLNPK.A								3						3
ACTA2 Actin, aortic smooth muscle Totals: Unique Peptides	14	1	1		1	3	2	8	27	2	3	5	3		40
NAT10 N-acetyltransferase 10	R.GDDEEWNEVLNKAGPNASIISLKSDKK.R	1		2		1		4		2					2
NAT10 N-acetyltransferase 10 Totals: Unique Peptides	1	1		2		1		4		2					2
INTS6 Isoform 1 of Integrator complex subunit 6	K.IESDRVIGSVG.K	1		1	1	1		4	1	1	1				3
INTS6 Isoform 1 of Integrator complex subunit 6 Totals: Unique Peptides	1	1		1	1	1		4	1	1	1				3
HIST1H1B Histone H1.5	K.ALAAGGYDVEK.N						1	1							
	K.ALAAGGYDVEKNNSR.I					1		1							
	R.KATGPPVSELITK.A					2	1	3							
	R.NGLSLAALKK.A					1		1							
HIST1H1B Histone H1.5 Totals: Unique Peptides	4					4	2	6							
NFKB1 Isoform 2 of Nuclear factor NF-kappa-B p105 subunit	R.RLEPVVSDAIYDSK.A	1	1			1	1	4		1	1		1		3
NFKB1 Isoform 2 of Nuclear factor NF-kappa-B p105 subunit Totals: Unique Peptides	1	1	1			1	1	4		1	1		1		3
HSPA5 HSPA5 protein	K.NQLTSNPENTVFDAK.R									1		1	2		4
	K.SQIFSTASDNQPTVTIK.V											2	1		3
	K.TKPYIQVDIGGGQTK.T											1			1
	K.VTHAVVTVPAYFNDAQR.Q											1			1
	K.VYEGERPLTK.D					1	1	2		1		1	1		3
	R.GVPQIEVTFEIDVNGILR.V											2			2
	R.ITPSYVAFTPEGER.L					1		1	1	2		1	1		5
	R.NELESYAYSLK.N												1		1
	R.TWNDPSVQQDIK.F					1	1	2	1			1	1		3
HSPA5 HSPA5 protein Totals: Unique Peptides	9					3	2	5	2	4		10	7		23
HSPA8 Isoform 1 of Heat shock cognate 71 kDa protein	K.NSLESYAFNMK.A					1		1							
	R.TTPSYVAFTDTER.L	1				1		2		1		2	1		4
HSPA8 Isoform 1 of Heat shock cognate 71 kDa protein Totals: Unique Peptides	2	1				2		3		1		2	1		4
TCF4 Isoform SEF2-1A of Transcription factor 4	R.SRSSNNDDEDLTPEQKAEREK.E					2		2	1	2					3
TCF4 Isoform SEF2-1A of Transcription factor 4 Totals: Unique Peptides	1					2		2	1	2					3
PVRL3 Isoform 1 of Poliovirus receptor-related protein 3	Q.DELDSYPDSVK.K			3		1		4	2	2	3	1			8
PVRL3 Isoform 1 of Poliovirus receptor-related protein 3 Totals: Unique Peptides	1			3		1		4	2	2	3	1			8
MLLT6 Protein AF-17	G.SMGGGGSGFISGRRSR.S		1					1	1	1			1		3
MLLT6 Protein AF-17 Totals: Unique Peptides	1		1					1	1	1			1		3
RTEL1 Isoform 4 of Regulator of telomere elongation helicase 1	R.AQPVLDPTGNFPDALDQLCGST.S				1			1		2					2
RTEL1 Isoform 4 of Regulator of telomere elongation helicase 1 Totals: Unique Peptides	1				1			1		2					2
MYL6B Myosin light chain 6B	K.ILYSQCGDVMR.A								1						1
	R.ALGQNPTNAEVLK.V					1	1	2	3	1	1	1	1		7
MYL6B Myosin light chain 6B Totals: Unique Peptides	2					1	1	2	4	1	1	1	1		8
ACTB Actin, cytoplasmic 1	E.TFNTPAMYVAIQAVLSLYASGR.T								1						1
	K.DLYANTVLSGGTTMYPGIADR.M								4						4
	K.MTQIMFETFNTPAMYVAIQAVLSLYASGR.T								2						2
	K.QEYDESGPSIVHR.K								1						1
	K.YSVWIGGSILASLSTFQQMWISKQEYDESGPSIVHR.K								2						2
	R.FRCPEALFQPSFLGMESCGIHETTFNSIMK.C								1						1
	R.GYSFTTTAER.E								2	1			1		4
	R.KDLYANTVLSGGTTMYPGIADR.M								2						2
	R.KYSVWIGGSILASLSTFQQMWISKQEYDESGPSIVHR.K								1						1
	R.TTGIVMDSGDGVTHTVPIYEGYALPHAILR.L								3						3
	R.VAPEEHPVLLTEAPLNPK.A					1		1	3		2	2	2		9
ACTB Actin, cytoplasmic 1 Totals: Unique Peptides	11					1		1	22	1	2	2	3		30
MYH9 Isoform 1 of Myosin-9	K.AKQTLENERGELANEVK.V								2						2
	K.ALELDSNLYR.I								1						1
	K.ANLQIDQINTDLNLER.S								3						3
	K.DDVGKSVHELEK.S								1						1
	K.DFSALESQLQDTQELLQEENR.Q								7				1		8
	K.DFSALESQLQDTQELLQEENRQK.L								2						2
	K.DLEGLSQR.H								1						1

Additional File 1: Mass Spectrometry Data For Pyk2 Complexes in U373

	Condition	Pyk2						Pyk2 Total	US28&Pyk2						US28&Pyk2 Total
Description	Peptide Sequence	0	5	10	15	30	60		0	5	10	15	30	60	Min post-stimulation
	K.FDQLLAEEK.T								1				1		2
	K.HEAMITDLEER.L								1						1
	K.HSQAVEELAEQLEQTK.R								2						2
	K.HSQAVEELAEQLEQTKR.V								3						3
	K.IAQLEEQLDNETK.E								3						3
	K.IAQLEEQLDNETKER.Q								2						2
	K.KANLQIDQINTDLNLER.S								2						2
	K.KFDQLLAEEK.T								3						3
	K.KRHEMPPHIYAITDTAYR.S								2						2
	K.KVEAQLQELQVK.F								4						4
	K.LKNKHEAMITDLEER.L								3						3
	K.LKSMEAEMIQLQEELAAAER.A								2						2
	K.LQKDLEGLSQR.H								2						2
	K.LQVELDNVTGLLSQSDSK.S												1		1
	K.LRLEVNLQAMK.A								1						1
	K.LTKDFSALESQLQDTQELLQEENR.Q								3						3
	K.LTKDFSALESQLQDTQELLQEENRQK.L								2						2
	K.MQQNIQELEEQLEEEESAR.Q								3						3
	K.NFINNPLAQADWAAK.K								1						1
	K.NKHEAMITDLEER.L								4						4
	K.NMDPLNDNIATLLHQSSDKFVSELWKDVDR.I								2						2
	K.QKKFDQLLAEEK.T								1						1
	K.QTLENERGELANEVK.V								2						2
	K.RDLGEELEALKTELEDTLDSTAAQQELR.S								2						2
	K.RQAQQERDELADEIANSSGK.G								1						1
	K.RQLEEAEEEAQR.A								2						2
	K.RQLEEAEEEAQRANASR.R								1						1
	K.SMEAEMIQLQEELAAAER.A								5						5
	K.TDLLLEPYNK.Y								1						1
	K.TQLEELEDELQATEDAK.L								3						3
	K.VAAYDKLEK.T								1						1
	K.VGRDYVQK.A								1						1
	K.VKVNKDDIQK.M								1						1
	K.VSHLLGINVTDFTR.G								3						3
	K.YLYVDKNFINNPLAQADWAAK.K								1						1
	R.ALEEAMEQK.A								2						2
	R.ALEEAMEQKAELER.L								2						2
	R.ALEQQVEEMKTQLEELEDELQATEDAK.L								5		2				7
	R.ALEQQVEEMKTQLEELEDELQATEDAKLR.L								3						3
	R.ALEQQVEEMKTQLEELEDELQATEDAKLRLEVNLQAMK.A								3						3
	R.ASREEILAQAKENEK.K								3						3
	R.ASREEILAQAKENEKK.L								1						1
	R.DLGEELEALKTELEDTLDSTAAQQELR.S								8	1	3	2	2		16
	R.DLQGRDEQSEEKKK.Q								1						1
	R.EEILAQAKENEK.K								1						1
	R.ELEDATETADAMNR.E								4						4
	R.EMEAELEDERK.Q								1						1
	R.EVSSLKNK.L								1						1
	R.FLSNGHVTIPGQQDKDMFQETMEAMR.I								2						2
	R.HEMPPHIYAITDTAYR.S								2						2
	R.IAQLEEELEEEQGNTELINDR.L								3						3
	R.IAQLEEELEEEQGNTELINDRLK.K								2						2
	R.IIGLDQVAGMSETALPGAFK.T								5						5
	R.INFDVNGYIVGANIETYLLEK.S								3						3
	R.KKVEAQLQELQVK.F								4						4
	R.KLEGDSTDLSDQIAELQAQIAELKMQLAKKEEELQAALAR.V								3						3
	R.LEVNLQAMK.A								2				1		3
	R.LQQELDDLLVDLDHQR.Q								4						4
	R.NTDQASMPDNTAAQK.V								1						1
	R.QAQQERDELADEIANSSGK.G								2						2
	R.QAQQERDELADEIANSSGKGALALEEK.R								1						1
	R.QKHSQAVEELAEQLEQTK.R								1						1
	R.QKHSQAVEELAEQLEQTKR.V								1						1
	R.QLEEAEEEAQR.A								2				1		3
	R.TEMEDLMSSK.D								2						2
	R.TEMEDLMSSKDDVGK.S								2						2
	R.TFHIFYYLLSGAGEHLK.T								2						2
	R.TFHIFYYLLSGAGEHLKTDLLLEPYNK.Y								1						1
	R.TFHIFYYLLSGAGEHLKTDLLLEPYNKYR.F								2						2
	R.TVGQLYKEQLAK.L								1						1
	R.VISGVLQLGNIVFK.K								3						3
	R.VISGVLQLGNIVFKK.E								2						2
**MYH9 Isoform 1 of Myosin-9 Totals: Unique Peptides**	**79**								176	1	5	2	7		191
MYL6B;MYL6 Isoform Non-muscle of Myosin light polypeptide 6	K.DQGTYEDYVEGLR.V								2						2
	K.NKDQGTYEDYVEGLR.V								4						4
	K.VLDFEHFLPMLQTVAK.N								4	1	2	1	3		11
**MYL6B;MYL6 Isoform Non-muscle of Myosin light polypeptide 6 Totals: Unique Peptides**	**3**								10	1	2	1	3		17
ACTA1 Actin, alpha skeletal muscle	K.DLYANNVMSGGTTMYPGIADR.M								3						3
	K.IWHHTFYNELR.V								1				1		2
	K.YPIEHGIITNWDDMEKIWHHTFYNELR.V								8		1				9
	R.KDLYANNVMSGGTTMYPGIADR.M								2						2
**ACTA1 Actin, alpha skeletal muscle Totals: Unique Peptides**	**4**								14		1		1		16
TPM1 tropomyosin 1 alpha chain isoform 2	K.AEADVASLNR.R								1						1
	K.LDKENALDRAEQAEADKKAAEDR.S								2						2
	K.LEEAEKAADESER.G								2						2
	K.SIDDLEEK.V								1						1
	K.VAHAKEENLSMHQMLDQTLLELNNM.-								1						1
	R.IQLVEEELDR.A								3				1		4
	R.IQLVEEELDRAQER.L								1						1
	R.KLVIIESDLER.A								1						1
	R.LATALQKLEEAEK.A								1						1
	R.LATALQKLEEAEKAADESER.G								2						2
	R.RIQLVEEELDR.A								1						1
**TPM1 tropomyosin 1 alpha chain isoform 2 Totals: Unique Peptides**	**11**								16				1		17
MGC42105 Serine/threonine-protein kinase NIM1	V.PVMMLPDPKERDLKK.G											1			1
**MGC42105 Serine/threonine-protein kinase NIM1 Totals: Unique Peptides**	**1**											1			1
USP6 Oncogene	Y.KHSSKLIDRVYK.G											1			1
**USP6 Oncogene Totals: Unique Peptides**	**1**											1			1
ABI2 Isoform 1 of Abl interactor 2	V.PSPTRNMAPSQQSPVR.T												1		1
**ABI2 Isoform 1 of Abl interactor 2 Totals: Unique Peptides**	**1**												1		1
CPNE5 Copine-5	L.IITDGVISDMAQTKEAIVNAAKLPMS.I													1	1
**CPNE5 Copine-5 Totals: Unique Peptides**	**1**													1	1
